# *anaklasis*: a compact software package for model-based analysis of specular neutron and X-ray reflectometry data sets

**DOI:** 10.1107/S1600576721009262

**Published:** 2021-10-20

**Authors:** Alexandros Koutsioubas

**Affiliations:** a Jülich Centre for Neutron Science (JCNS) at Heinz Maier-Leibnitz Zentrum (MLZ), Forschungszentrum Jülich GmbH, Lichtenbergstrasse 1, 85748 Garching, Germany

**Keywords:** X-ray reflectometry, neutron reflectometry, fitting software

## Abstract

*anaklasis* constitutes a set of open-source Python scripts that facilitate a range of specular neutron and X-ray reflectivity calculations, involving the generation of theoretical curves and the comparison/fitting of interfacial model reflectivity against experimental data sets. The primary focus of the software is twofold: on one hand to offer a more natural framework for model definition, requiring minimum coding literacy, and on the other hand to include advanced analysis methods that have been proposed in recent work. Particular attention is given to the ability to co-refine reflectivity data and to the estimation of model-parameter uncertainty and covariance using bootstrap analysis and Markov chain Monte Carlo sampling. The compactness and simplicity of model definition together with the streamlined analysis do not present a steep learning curve for the user, an aspect that may accelerate the generation of reproducible, easily readable and statistically accurate reports in future neutron and X-ray reflectivity related literature.

## Abstract 2

A new software package (*anaklasis*) for model-based analysis of specular neutron and X-ray reflectivity is introduced. Key features include a user-friendly compact interfacial model definition scheme and a complete set of methods for co-refining data and estimating parameter uncertainty.

## 1. Introduction

Specular neutron and X-ray reflectometry (NR and XRR) are established experimental techniques for the investigation of the structure of interfaces at the sub-nanometre scale (Penfold & Thomas, 1990 ▸; Daillant & Gibaud, 2008 ▸; Born & Wolf, 2019 ▸; Heavens, 1955 ▸). XRR and NR experiments are performed by shining collimated X-ray or neutron beams at interfaces and by registering the specularly reflected radiation intensity (*R*) as a function of momentum transfer ( 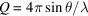 , where θ is the incidence angle and λ is the wavelength of the incident radiation). Owing to the relatively short wavelengths of X-rays and cold neutrons, the presence of nanometre-scale films at interfaces gives rise to interference effects that modulate the observed reflectivity. In this sense the experimentally measured reflectivity *R*(*Q*) can be related to the nanometre-scale features of an interface.

Depending on the nature of the used radiation, calculation of *R*(*Q*) for a given scattering length density (sld) profile (where sld is defined as the number-density-weighted nanometre-scale average of the scattering lengths of the layer’s atomic constituents) is routinely performed using the Schrödinger or Helmholtz equations (derived from Maxwell’s equations) and by applying appropriate boundary conditions of wavefunction continuity and momentum conservation or of tangential electric and magnetic field component continuity, respectively, at layer boundaries. However, for the solution of the inverse problem [*i.e.* recovering the sld profile from *R*(*Q*)], complications arise mainly because only the amplitudes of reflected electromagnetic waves or neutron wavefunctions are measured during an experiment, thus leading to the loss of phase information (the phase problem).

Several different ‘model-independent’ approaches for the reconstruction of interfacial structure from reflectivity measurements have been reported, based either on experimental (Majkrzak & Berk, 1998 ▸; Majkrzak *et al.*, 2003 ▸, 2000 ▸) or on numerical/stochastic methods (Pedersen, 1992 ▸; Hohage *et al.*, 2008 ▸; Kunz *et al.*, 1993 ▸; Zhou & Chen, 1993 ▸; Koutsioubas, 2019 ▸). However, the vast majority of investigations in the literature rely traditionally on model-dependent refinements, where the interface is modelled as a stratified medium (succession of slabs) with prior knowledge about the system being embodied in the bounds and relations between the sld, thickness and roughness of each slab. Driven by that, several software packages have been developed addressing the needs of refining interfacial-model parameters with respect to experimental data, with some of them specifically adapted to different experimental scenarios, like polarized neutron data, contrast-variation data sets and NR/XRR co-refinement. (For a fairly complete and historic list of developed reflectivity software the reader is referred to https://www.reflectometry.org/information/software/.)

Notably, a subset of these programs has found widespread use by the scientific community working with NR and XRR. Among these, *GenX* (Björck & Andersson, 2007 ▸) is a Python graphical user interface (GUI) and script-based program that permits the execution of elaborate refinements by the expert user. Additionally, with *GenX* the use of differential evolution minimization has been introduced to reflectivity software. The *Motofit* (Nelson, 2006 ▸), *RasCAL* (Hughes, 2019 ▸) and *Aurore* (Gerelli, 2016 ▸) programs mainly address the case of co-refining multiple solvent-contrast data from solid/liquid and air/liquid interfaces. Finally, *refnx* (Nelson & Prescott, 2019 ▸) and *Refl1D* (Kienzle *et al.*, 2011 ▸) are powerful packages that have introduced the use of Markov chain Monte Carlo (MCMC) sampling for investigating parameter uncertainty and covariance.

An important aspect of reflectivity analysis software is the adopted way of defining the interfacial model, *i.e.* sld, thickness and roughness of the layers, and their relation with other defined parameters. GUI-based spreadsheet-like input of parameters for each layer offers simplicity but tends to be restrictive for the definition of elaborate layer models. On the other hand, script-based model definition, while being in principle flexible, may be complicated for the new user since it requires writing package and computer language specific code (classes, functions *etc.*) for the definition of layer structures and constraints between parameters. Additionally, the model definition has to address issues related to the way that co-refinement of data from contrast-variation series or different neutron-beam polarizations is handled.

In the present article we introduce a new software package under the name *anaklasis* [ 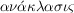 (anaklasis) in ancient Greek means reflection], where we combine an intuitive hierarchical list-based type of input with the flexibility typically found in script-based software, in a way that requires minimal coding literacy from the user. (Essentially, the only coding skill that is required concerns the definition and basic manipulation of lists in Python.) The main novelty is the ability to define layer features (sld, thickness, roughness *etc.*) directly as symbolic mathematical expressions involving parameters. This aspect also extends to the definition of constraints between parameters in the form of inequalities. The resulting compact model definition simplifies reporting data-refinement workflows in publications and creates a more natural framework for new model definition, which usually represents the main time bottleneck in analysing reflectivity data. The above-mentioned characteristics of the package do not come at the expense of advanced features such as the ability to handle mixed-area models, co-refine data, or estimate model-parameter uncertainty and covariance.

*anaklasis* includes the following key features:

(1) Compact and flexible model definition, based on the creation of Python lists that contain layer data as numerical values and/or as *SymPy* (Meurer *et al.*, 2017 ▸) symbolic expressions that involve parameters.

(2) Co-refinement of data (non-spin-flip polarized neutron data sets, contrast-variation data sets) through the use of ‘multi-parameters’.

(3) Straightforward constraint definitions involving expressions between model parameters.

(4) Use of the robust differential evolution *SciPy* (Virtanen *et al.*, 2020 ▸) minimizer.

(5) Effortless estimation of parameter uncertainty and covariance through MCMC or bootstrap statistics.

(6) Easily readable output and ready-to-publish graphical output.

(7) Open-source code under the GPL v3 licence that may be installed on all major platforms (Windows/macOS/Linux).

In the following sections we outline the interfacial model definitions and the methods used for reflectivity calculations, data refinement and statistical analysis. Then, through a set of representative examples, we validate the abilities of the software package and showcase that it may address the vast majority of refinement scenarios encountered when analysing NR and XRR data.

## 2. Methods

*anaklasis* is written in Python 3 with Fortran 90 extensions for the computationally intensive reflectivity calculations.^1^ These are performed using the Abelès matrix method (Heavens, 1955 ▸), where layer roughness is taken into account using the Névot–Croce approximation (Névot & Croce, 1980 ▸). The ref submodule contains three callable functions, ref.calculate for generating theoretical reflectivity curves, ref.compare for comparison of experimental data with theoretical curves and ref.fit for refinement of experimental data against a defined model. Execution of the program takes place by defining the interfacial model and instrumental parameters as lists in a simple Python script and by passing them as arguments to the desired function.

### 2.1. Model definition

Stratified-layer interfacial system definition is accomplished by the creation of a hierarchical list structure. In order to cover the general case where no single laterally uniform structure is present at the interface,^2^ the system is defined as a list that may contain multiple models (patches) with an associated surface coverage that contribute incoherently to the calculated reflectivity. Note that in practice, in most cases, a single patch with a surface coverage equal to unity needs to be defined. Each model is a list containing an arbitrary number of layers (slabs). In turn, layers are also represented as lists composed of six elements, *i.e.* real/imaginary part of the sld, thickness, roughness, solvent volume fraction^3^ and description (see SI0 in the supporting information for a pictorial representation).

The elements of the layer list (except for the description) can be either numerical values^4^ or *SymPy* mathematical expressions. *SymPy* is a versatile package for symbolic computation which, besides basic algebra, permits the construction of expressions containing sums, derivatives and integrals and/or a variety of functions like trigonometric, logarithmic and exponential. Expressions may include the layer number [from 0-fronting to (*N* + 1)-backing medium] and an arbitrary set of user-defined global parameters, whose values and descriptions are inserted in a separate list. Model definition is accompanied by information related to the instrumental parameters. These include δ*Q*/*Q* resolution, incoherent background and a scale factor in the case of non-normalized reflectivity.^5^ As will be shown in the coming sections, this mode of input coupled with symbolic mathematics provides enough flexibility for defining rather complex models.

### 2.2. Data-refinement-related definitions

For experimental-data refinement with ref.fit, model definition is the same as when we perform theoretical reflectivity calculations with the ref.calculate and ref.compare functions. However, we additionally need to specify which of the defined parameters are fixed and which are free to vary within specified bounds. For this purpose, two numerical values are specified for each global parameter, which represent either the min/max bound of a uniform distribution or the mean and standard deviation in the case of a normally distributed parameter. An identical min/max value or a zero standard deviation signifies a fixed parameter.

In order to treat the case of co-refinement of an arbitrary number of *M* input curves (NR and/or XRR) with the same model, on top of global parameters we also introduce multi-parameters, *i.e.* parameters that may adopt a different value or set of bounds for each input curve. Their definition is similar to global parameters, the difference being that *M* min/max or mean/standard deviation pairs have to be specified, each one corresponding to an input experimental curve. Multi-parameters together with global parameters can be used in the symbolic expressions inserted in the layer list.

As well as the definition of expected values for specific parameters, prior knowledge about the system under study might require application of constraints that need to be expressed as inequalities involving defined global and multi-parameters. *anaklasis* supports straightforward definition of such constraints as *SymPy* expressions. Application of these concepts will be the matter of many of the examples in the next section.

### 2.3. Types of experimental data sets

In most cases, reflectivity data are stored in a two-, three- or four-column format corresponding to *Q*, *R*(*Q*), δ*R*(*Q*) and δ*Q*. Depending on the type of instrument, experimental error δ*R*(*Q*) and/or resolution information δ*Q* might be missing or considered as unreliable (common for XRR). When experimental error information is missing, a refinement can be performed without parameter uncertainty estimation. On the other hand, if δ*Q* (full width at half-maximum of a Gaussian approximation to the instrument resolution function) is not present, meaning that point-wise smearing using Gaussian convolution cannot be performed, the user may define a constant δ*Q*/*Q* that is applied to the entire *Q* range.

*anaklasis* supports the input of two-, three- or four-column ASCII data containing footprint-corrected reflectivity data with *Q* and δ*Q* in units of Å^−1^ or nm^−1^. In future versions of the program we intend to support the file format that will be defined by ORSO.

### 2.4. Minimization and parameter uncertainty estimation

Depending on the type of data input [*i.e.* availability of δ*R*(*Q*)] and on user choice for fitting on the linear [*R*(*Q*)] or logarithmic [log_10_ *R*(*Q*)] scale, during data refinement the following figure of merit (FOM) gets minimized with respect to the set of free parameters α:

(*a*) *R*(*Q*) with errors: 

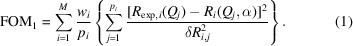




(*b*) log_10_ *R*(*Q*) with errors: 

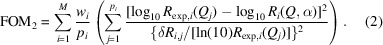

[The expression in the denominator inside the sum comes from error propagation theory, where δ(log_10_ *R*)^2^ = [δ*R*/*R*ln(10)]^2^.]

(*c*) *R*(*Q*) no errors (with 1/*R* weighting): 

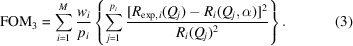




(*d*) log_10_ *R*(*Q*) no errors: 

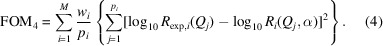




Here, *w*_*i*_ is the fit weight of the input curve *i*, and the subscript *j* runs over the number of points (*p*_*i*_) of each data set.

Minimization is performed using the differential evolution algorithm (Storn & Price, 1997 ▸) available in *SciPy* (Virtanen *et al.*, 2020 ▸), which has proven to be a robust minimizer for reflectivity data (Björck & Andersson, 2007 ▸) that avoids local minima. After a successful minimization and if the experimental error d*R*(*Q*) is available, there are three ways of estimating the uncertainty of the model’s parameters:

(1) The fastest method, although sometimes prone to numerical instabilities, is through a numerical estimation (*numdifftools* package; D’Errico, 2006 ▸), near the FOM minimum, of the diagonal elements *H*_*kk*_ of the Hessian matrix, which are the second-order partial derivatives of the reduced-χ^2^ ( 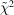 ) with respect to each free parameter. Then the standard deviation of the *k*th parameter is given by (Gerelli, 2016 ▸) 

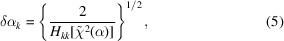

where 

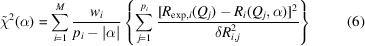

and |α| is the number of free parameters.

(2) The second and quite computationally demanding option, originally implemented in the program *Aurore* (Gerelli, 2016 ▸), is based on the bootstrap method, where each experimental curve is replicated *K* times (*K* = 1000 in *anaklasis*) by replacing each [*R*(*Q*), δ*R*(*Q*)] data point with [*R*(*Q*) + δ_rand_, δ*R*(*Q*)], where δ_rand_ is a random number belonging to a normal distribution with a mean equal to zero and standard deviation equal to δ*R*(*Q*). Then by repeating independently the minimization for all *K* generated data sets, we calculate both the mean and standard deviation of each free parameter.

(3) The last and probably most efficient method is based on a Bayesian MCMC sampling of the system that examines the posterior probability of free parameters, which is proportional to the product of the prior probability and the likelihood. MCMC was initially implemented for reflectivity analysis in *Refl1D* (Kienzle *et al.*, 2011 ▸), but here we closely follow the methodology proposed in *refnx* (Nelson & Prescott, 2019 ▸). MCMC sampling in *anaklasis* is performed using *emcee* (Foreman-Mackey *et al.*, 2013 ▸) and by assuming that the measurement uncertainties δ*R*(*Q*) are normally distributed. Automatically, an initial run generating a 500-sample chain (*i.e.* sets of parameters compatible with data and prior information) is used for estimating the ‘integrated autocorrelation time’ (τ). The estimate is used for discarding 10τ samples and for performing an actual production run for at least 60τ samples.

Note that both bootstrap and MCMC methods, except for uncertainty estimation, give us the ability to draw a correlation corner plot of all free parameters, where we may visually identify correlations between free parameters and any probable distribution multimodality or asymmetry close to imposed parameter bounds that may indicate a required revision of the initially considered bound.

## 3. Reflectivity calculation and refinement examples

In order to familiarize readers with aspects of interfacial system definition in *anaklasis*, in this section we present two examples of reflectivity calculations. Then we move to data-refinement examples that represent frequently encountered cases in NR and XRR research.

### 3.1. Two simple layers

**Figure 1:**
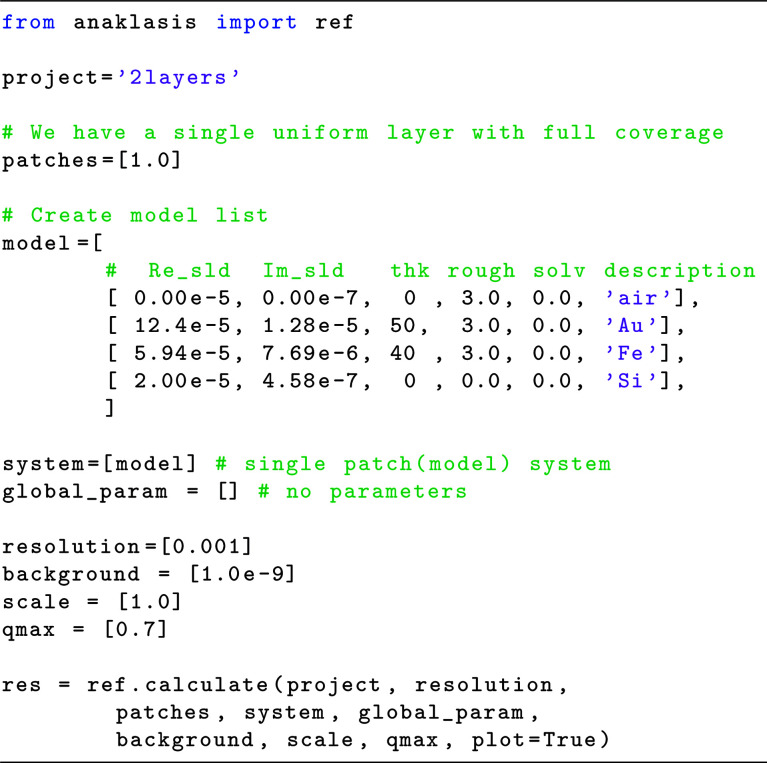
Python code for XRR calculations of an Fe/Au film pair at the Si/air interface.

Let us consider the relatively simple case of an XRR experiment at the air/solid (Si) interface, with the presence of two thin layers, a 40 Å Fe and a 60 Å Au film. We also assume a roughness for all layers equal to 3 Å. The instrumental resolution δ*Q*/*Q* and background have typical values for synchrotron XRR. The Python code for calculating the reflectivity of such a model is presented in Fig. 1 ▸.

**Figure 2:**
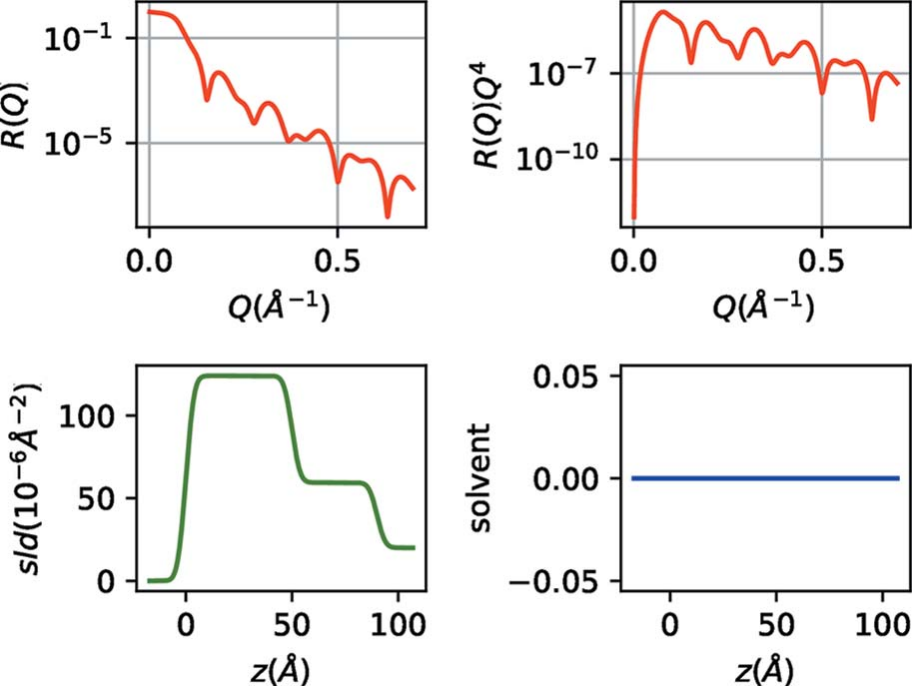
XRR and sld/solvent volume fraction profiles for the Fe/Au film pair on Si.

The code with its brief comments is almost self-explanatory in this simple case of a single model (patch) interface, where we just fill layer lists with the numerical values of each parameter as done in GUI-based programs. By definition, the roughness value of layer *i* refers to the actual roughness between layers *i* and *i* + 1. Note that the fronting and backing media have infinite thickness, and in the scripts we use the convention of inserting a zero value (although using any other numerical value will not influence the calculations). The corresponding graphical output (Fig. 2 ▸) includes the theoretical reflectivity in *R*(*Q*) and *R*(*Q*)*Q* ^4^ representation and the sld and solvent volume fraction profiles. In the current example and since no liquid media are present, the solvent volume fraction profile is not relevant. Note that if experimental data are available, and we want to compare the theoretical reflectivity and access the χ^2^, we just need to specify the input data file and call the ref.compare function at the end of the script (see the related example for a supported lipid bilayer in the supporting information SI1).

### 3.2. Nanoparticle islands on a substrate

We now pass to a more elaborate example. We consider an NR measurement at a solid (Si)/liquid (D_2_O) interface that is 70% covered by millimetre-sized islands (patches) of close-packed polystyrene (PS) spherical nanoparticles having a diameter *D* = 150 Å. Because the island lateral size is orders of magnitude larger than the typical coherence length of a neutron reflectometer, the total reflectivity is given by the weighted sum of contributions from the two models, *i.e.* Si/D_2_O and Si/PS/D_2_O. Model definition for Si/D_2_O is straightforward, but for the Si/PS/D_2_O system we need an expression for the volume fraction of the nanoparticles normal to the substrate. For spherical nanoparticles of diameter *D* on a substrate, the volume fraction is given by ϕ(*z*) = (4*A*/*D* ^2^)(*Dz* − *z* ^2^), where *A* is the volume fraction in the middle of the layer and for close packing *A* ≃ 0.91. So for the volume fraction of the solvent (D_2_O) in the nanoparticle layer we arrive at the expression

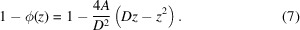




**Figure 3:**
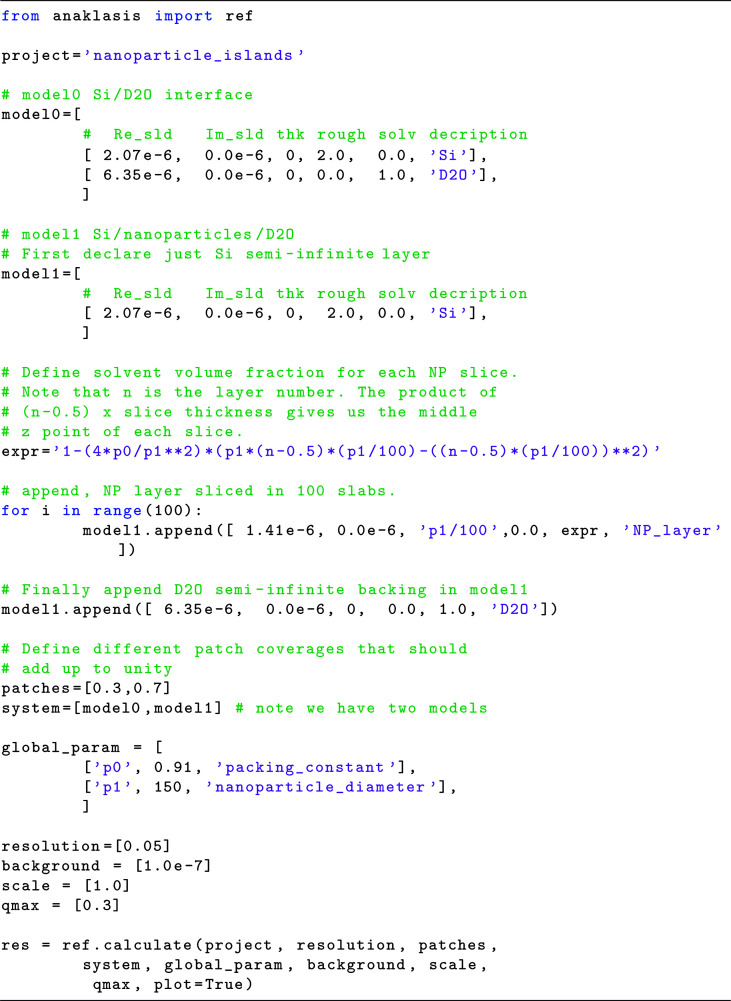
Python code for NR calculations of PS nanoparticle islands at the Si/D_2_O interface.

**Figure 4:**
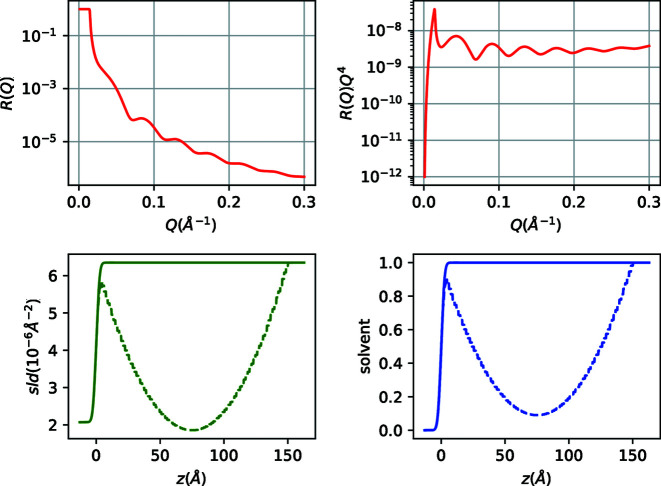
NR and sld/solvent volume fraction profiles for PS nanoparticle islands at the Si/D_2_O interface. Full lines represent Si/D_2_O and dashed lines Si/nanoparticles/D_2_O profiles.

By slicing the nanoparticle layer into 100 slabs of *D*/100 thickness, we construct the model as described in the commented code in Fig. 3 ▸. The corresponding output is plotted in Fig. 4 ▸.

We define the nanoparticle layer with a for loop as a succession of 100 1.5 Å-thick slices, while we use an algebraic expression for the solvent volume fraction [equation (7)] that includes two defined parameters (*p*_0_ → *A*, *p*_1_ → *D*) and the integer number *n* of each slice. This type of model building is particularly useful when we work with multilayers, where we can stack multiple layer structures using a for loop. Related examples concerning a phospholipid multilayer and a bimodal polymer brush can be found in the supporting information [SI2 and SI3; additional related literature: Anastassopoulos *et al.* (2013)].

Our calculation did not include possible polydispersity of the nanoparticles. If we want to take this into consideration then in *anaklasis* we just need to modify equation (7) and use an additional parameter describing nanoparticle polydispersity. Assuming that the nanoparticle diameter is distributed normally with a standard deviation σ_*D*_, we may rewrite the expression for the solvent volume fraction in the nanoparticle layer as 

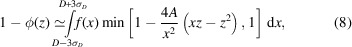

where 

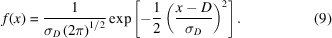

The code and related output for polydisperse nanoparticles following equation (8) can be found in the supporting information (SI4).

### 3.3. Polymer brush refinement

Some of the concepts of model building introduced in the last example will also be used here for the refinement of experimental NR data from a PS (*M*_w_ = 70 K) polymer brush at the quartz/d-toluene interface that have been acquired (Hiotelis *et al.*, 2008 ▸) at the now-decommissioned EROS time-of-flight reflectometer at the Laboratoire Léon Brillouin (Saclay). End-grafted linear polymer chains (brushes) due to a balance between entropic and steric interactions are expected from mean-field theory (Milner *et al.*, 1988 ▸) to form extended layers having a volume fraction profile of the form ϕ(*z*) = ϕ(0) − *Cz* ^*n*^. At sufficiently high grafting densities the exponent *n* is equal to 2 (parabolic profile). Setting the maximum brush-layer extension as *L*, and since ϕ(*L*) = 0, we may rewrite the above expression as ϕ(*z*) = ϕ(0)[1 − (*z*/*L*)^*n*^].

Using the same line of thought as in the previous example we may approximate the brush layer by a number of ‘thin’ slices having a solvent volume fraction

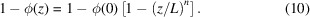




**Figure 5:**
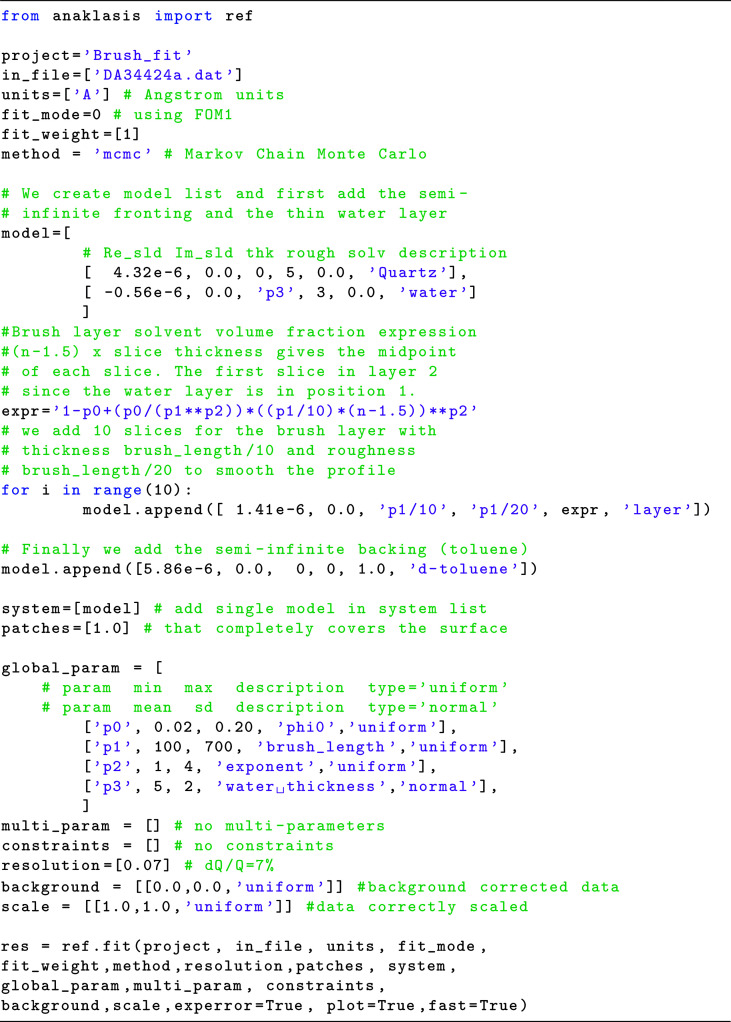
Python code for NR data refinement from a PS brush at the quartz/d-toluene interface.

Given that we want to fit the relevant experimental data, we define a set of global parameters that are left free to vary. These are the volume fraction at *z* = 0 [ϕ(0)], the brush length (*L*), the exponent (*n*) and the thickness of a thin H_2_O layer that is present at the interface. The parameters ϕ(0), *L* and *n* appear in the expression defining the solvent volume fraction profile of each brush layer slab. We assume that all of the parameters have a flat prior probability (uniform) to vary within the specified bounds, except for the thickness of the few-ångström-thick water layer that is defined as a normally distributed parameter given by its mean value and standard deviation. The corresponding code is given in Fig. 5 ▸.

**Figure 6:**
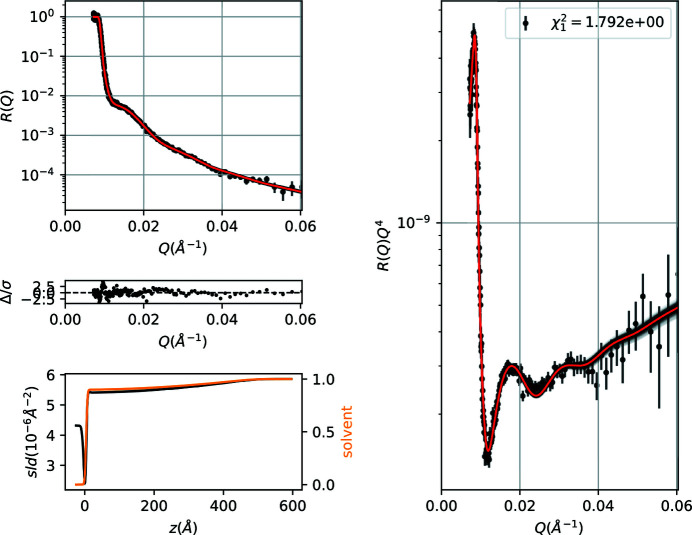
Fitted NR data and sld/solvent volume fraction profiles for a PS brush at the quartz/d-toluene interface.

**Figure 7:**
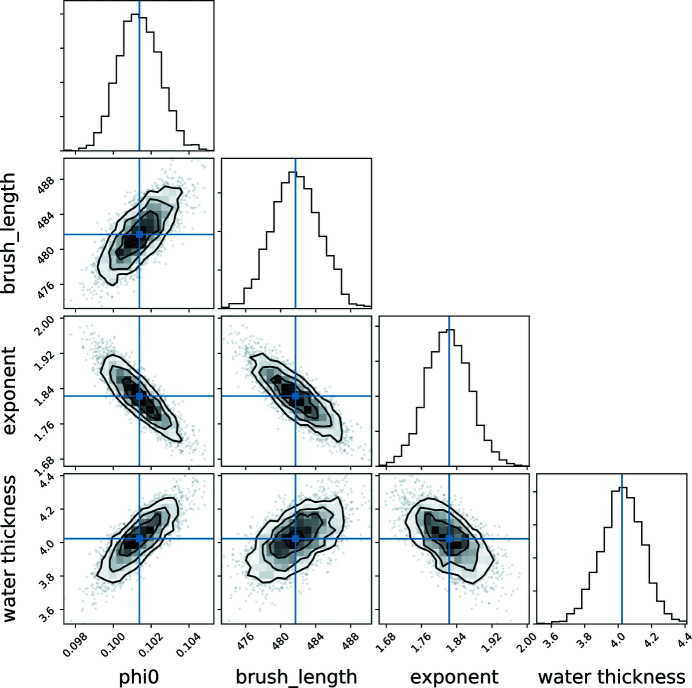
Corner plot of the free parameters during the PS brush NR data refinement. The panels on the diagonal show the 1D histogram for each model parameter obtained by marginalizing over the other parameters, with a blue line to indicate the mean value. The off-diagonal panels show 2D projections of the posterior probability distributions for each pair of parameters.

The data refinement gives a water-layer thickness 3.9 ± 0.1 Å, ϕ(0) = 0.10 ± 0.01, *L* = 480 ± 2.7 Å and *n* = 1.85 ± 0.05. Using MCMC sampling (or bootstrap) except for parameter uncertainty estimation, together with the fitted curves and sld/solvent profiles we also plot the corresponding 1σ confidence intervals (Fig. 6 ▸). Additionally, we obtain a corner plot of the free parameters that is informative about covariances or multi-modalities. In the present case (Fig. 7 ▸) the slight stretch in the 2D projections of the posterior probability distribution of parameter pairs suggests a moderate covariance between parameters.

### 3.4. Refinement of lipid bilayer in three-solvent contrasts

A type of NR data refinement that is encountered quite frequently concerns the concurrent fitting of reflectivity curves from solvent-contrast-variation series, a method that permits an overall reduction of modelling ambiguity (Fragneto *et al.*, 1995 ▸; Braun *et al.*, 2017 ▸; Wacklin, 2010 ▸). Supported phospholipid membranes at the solid/liquid interface represent an archetypical system that can be studied in this way, where the same structural model is used for fitting multiple curves and only the solvent contrast is varied. Here let us consider a three-contrast data set [D_2_O, Si-matched water (SMW) and H_2_O] of a dimyristoylphosphatidylcholine (DMPC) supported bilayer at the Si/water interface acquired on the Platypus neutron reflectometer (ANSTO) (James *et al.*, 2006 ▸) and distributed as an example with the package *refnx* (Nelson & Prescott, 2019 ▸).

We model the interface using a six-layer model as SiO_2_/thin water layer/inner lipid heads/inner lipid tails/outer lipid tails/outer lipid heads, where solvent may partially penetrate into each lipid layer. Given that the surface area per molecule (*A*_pm_) is the same for both lipid leaflets, the sld (not accounting for water penetration) and solvent volume fraction ϕ_solv_ of each of the four lipid layers are given by 

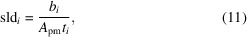




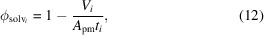

where *t* is the layer thickness and *b* and *V* are the corresponding scattering length and molecular volume, respectively.

**Figure 8:**
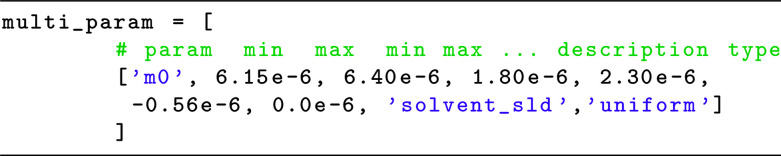
Python code for defining the multi-parameter list used in three-contrast NR phospholipid bilayer refinement.

In the supporting information (SI5) we present the commented code listing containing the lipid-bilayer model based on equations (11) and (12), where we additionally apply a set of constraints so that the solvent volume fraction always stays larger than zero during parameter refinement. This is accomplished by populating the constraint list with expressions of the type 1 − *V*_*i*_/(*A*_pm_ *t*_*i*_) > 0. Special mention needs to be made of how the solvent sld is handled for each input curve. We define a multi-parameter of the form shown in Fig. 8 ▸, where three min/max bound pairs, one for each contrast, are inserted. The use of the multi-parameter in expressions is the same as for global parameters, the only difference being that the bounds are specific for each input curve. Here we have chosen to use different min/max bound values for all three contrasts. This leaves the solvent sld as a free parameter, accounting for an imperfect solvent exchange during the measurement procedure.

**Figure 9:**
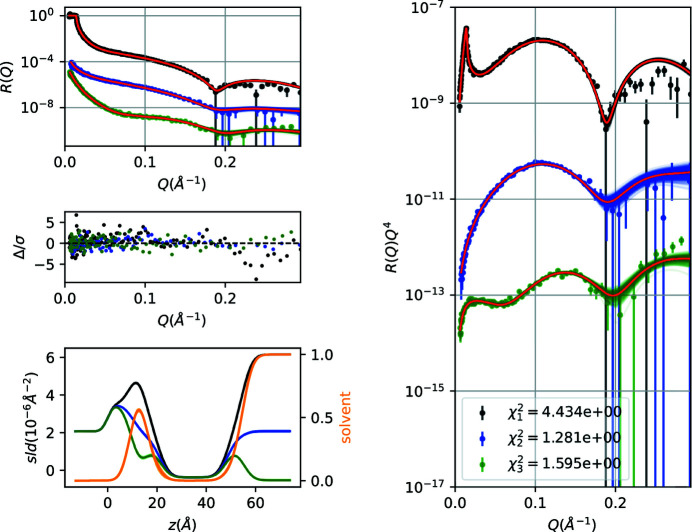
Co-refined NR data and sld/solvent volume fraction profiles for a supported DMPC bilayer at the Si/water interface. The reflectivity curves are systematically shifted on the vertical axis for clarity. Black, blue and green points correspond to D_2_O, SMW and H_2_O solvent contrasts.

The bilayer parameters (area per lipid, inner head thickness, outer head thickness, tail thickness, roughness) together with thin water layer thickness, solvent sld, background and scale of each curve add up in total to 16 free parameters. The data fit (Fig. 9 ▸) results in parameter values well within expectations from previous literature. The corner plot of free parameters (supporting information SI5) reveals a relatively strong correlation between tail thickness and area per lipid, as also found by Nelson & Prescott (2019 ▸)

In the supporting information (SI6) we include an even more characteristic example of contrast manipulation in NR, based on measurements acquired by Hollinshead *et al.* (2009 ▸) and thoroughly reanalysed by McCluskey *et al.* (2019 ▸, 2020 ▸), where for a distearoylphosphatidylcholine (DSPC) lipid monolayer at the air/water interface the contrasts of both the water and lipids are varied systematically. We co-refine seven different curves with a single structural model, highlighting the use of multi-parameters.

### 3.5. Polarized neutron reflectivity refinement

**Figure 10:**
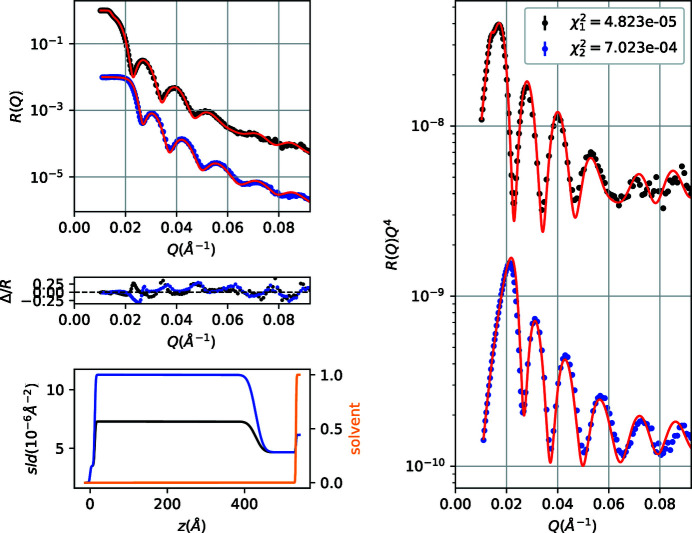
Co-refined PNR data for an Fe–Ni (∼400 Å)/Au (∼100 Å) film pair at the Si/water interface. The Python code for the fit of the data can be found in the supporting information (SI7). The reflectivity curves are systematically shifted on the vertical axis for clarity.

Multi-parameters in *anaklasis* also find a very convenient use in the case of another major application of NR, *i.e.* the study of magnetic thin films by non-spin-flip polarized NR (PNR) (Majkrzak *et al.*, 2006 ▸). For saturated magnetic thin films, the system is birefringent because the sld depends on the neutron polarization with respect to the magnetization. So in co-refinement of PNR data, a multi-parameter can be defined for setting the magnetic sld contribution depending on beam polarization. One such refinement of PNR (0.5 T applied magnetic field) from an Fe–Ni alloy/Au layer at the Si/D_2_O interface acquired on the MARIA reflectometer (MLZ) (Mattauch *et al.*, 2018 ▸) is shown in Fig. 10 ▸.

One may even combine in such a co-refinement both PNR and XRR data of the same sample, as described in an additional example in the supporting information (SI8).

## 4. Discussion

The initial motivation for developing *anaklasis* came from the observation that a usual bottleneck in the analysis of reflectivity results by users of neutron and X-ray facilities is related to the relative difficulty in implementing custom interfacial models in existing reflectivity software. When the system under study is simple and may be approximated by a succession of a few uniform layers, the use of GUI-based programs provides a convenient way of fitting experimental results. However, when an interfacial model that is based on intuition or previous knowledge about the system needs to incorporate analytical expressions and constraints between model parameters, GUI programs tend to be restrictive. Although there exist powerful reflectivity analysis packages (Nelson & Prescott, 2019 ▸; Kienzle *et al.*, 2011 ▸) where complicated models can be defined by writing package-specific code, we argue that *anaklasis* provides an alternative and more direct means of elaborate model definition, since functional dependences and inequality constraints between model parameters can be expressed in near natural mathematical language.

The adopted scheme of entering values or expressions into a Python list requires minimal coding literacy and produces a very compact representation of reflectivity data analysis. In principle, someone reading a script could deduce most details of calculations or refinements given the information on how global and multi-parameters work in *anaklasis*. On the other hand the use of *SymPy* expressions describing layer parameters and constraints, together with basic Python list manipulation, permits the definition of a very broad range of interfacial models, as showed by the given examples. Here we note that the only general use case that is not currently covered by *anaklasis* concerns spin-flip polarized reflectivity, and interested users are encouraged to use packages like *GenX* (Björck & Andersson, 2007 ▸) that explicitly treat such systems.

Experimental data input is flexible in terms of reflectivity data coming from different types of instruments (neutron or X-ray reflectometers, reporting or not experimental error and resolution). The option to weight the contribution of different curves in co-refinements or to use different figures of merit is offered as a way to remedy any detected bias towards high or low *Q* in the resulting fits. After model building and reaching an acceptable fit of the experimental data with the differential evolution minimizer, the user is provided with the option to proceed to an MCMC sampling or bootstrap analysis, obtaining a realisitic estimate of parameter uncertainty and possible covariance. The program output in the form of log files contains layer-by-layer detailed information, while graphical output as seen in previous sections summarizes the resulting sld profiles and the overall agreement between the model’s theoretical reflectivity and the experimental data. All functions return results in the form of multi-key dictionaries, so that scripts for batch calculations or result post-processing can be written. Furthermore, *anaklasis* can be incorporated into *Jupyter* notebooks (Kluyver *et al.*, 2016 ▸), aiding the production of elegant reports.

Future addition of features to the package will not break compatibility of already written scripts. For example, addition of a new type of bootstrap analysis based on the assumption of Poissonian statistics for δ*R*(*Q*) will be added as an additional keyword option for the argument method, thus not affecting past-developed refinement scripts. Envisioned capabilities for calculating reflectivity curves from molecular dynamics trajectories (Koutsioubas, 2016 ▸) will come in the form of new sub-modules. Additionally for the sake of reproducibility, a test script is provided for the core reflectivity calculations of the package so that all future versions may be tested before release.

While the incorporation of symbolic expressions in the interfacial system definition provides a more direct and natural framework for users, it also comes with relative performance penalties that accumulate as the number of defined layers and complexity of mathematical expressions increase. Although in the current version of *anaklasis* reflectivity calculations are not vectorized as in other packages (Nelson & Prescott, 2019 ▸; Kienzle *et al.*, 2011 ▸), the use of Fortran 90 extensions for the core reflectivity calculations and the fact that differential evolution and MCMC use all the available CPU cores (on a POSIX-compliant system, Linux/macOS) provide adequate speed of calculations during data refinements. Indicatively, the refinement of the three-solvent-contrast lipid bilayer data presented above, together with the MCMC sampling for the estimation of parameter uncertainty, takes less than 8 min on a four-core (eight-thread) modern mobile CPU running Linux. On the same machine, the presented full refinement of polymer brush data takes about 90 s, and bootstrap analysis close to 45 min.

Installation of the program requires the *NumPy* package (Harris *et al.*, 2020 ▸) and the presence of a Fortran compiler like *gfortran*. Since installation of a Fortran compiler on Windows might pose difficulties, a package with pre-compiled extensions is also provided for Windows 10. (The current version of the package also provides a slower pure Python calculation engine, thus not requiring an installed Fortran compiler.) All other required packages are handled automatically by the installation script. The program output includes a list of all packages used during calculations, together with their versions. *anaklasis* is released under the GPL v3 licence and all other dependencies are released under open-source licences. The source code, documentation and example library (examples are provided in the form of both scripts and annotated Jupyter notebooks) of the project are held in GitHub (https://github.com/alexandros-koutsioumpas/anaklasis) and users are encouraged to contribute interfacial models and refinement scripts that can be integrated into the examples library. Finally, an option to run *anaklasis Jupyter* notebooks in the cloud through the *Binder* (https://mybinder.org) project is offered, thus allowing users to perform analysis of reflectivity data using a web browser and without installing the software locally.

## 5. Conclusions

*anaklasis* is a new open-source tool for the analysis of XRR and NR data with a simple and compact interfacial model-definition method, providing advanced features for data refinement, including MCMC and bootstrap analysis. Its smooth learning curve may both accelerate treatment of data and aid in the reportability and reusability of published reflectivity results.

## Supplementary Material

Document containing examples of the use of the anaklasis package. DOI: 10.1107/S1600576721009262/in5053sup1.pdf

## Footnotes

1 The validity of reflectivity calculations performed by the package is checked periodically using the Open Reflectivity Standards Organization (ORSO; https://www.reflectometry.org) validation tests (https://github.com/reflectivity/analysis).

2 Here we refer to interfaces composed of different layer structures with lateral dimensions much larger than the coherence length of the reflected beam.

3 This applies in cases where a liquid is used as the fronting or backing medium.

4 All used units are in Å for length and Å^−2^ for sld.

5 Although in *anaklasis* a scale factor is provided as a parameter that may be varied in data refinements, it is in general suggested to avoid the use of ill-normalized data in model refinements, since they may lead to a biased structural model.
