# Supplementary material for: anaklasis: a compact software package for model-based analysis of specular neutron and X-ray reflectometry data sets
Source: J Appl Crystallogr. 2021 Oct 20;54(Pt 6):1857–66. doi: 10.1107/S1600576721009262 (PMC8662969; doi:10.1107/S1600576721009262)
Supplement: Supplementary file 1 [file j-54-01857-sup1.pdf]

JOURNAL OF
APPLIED
CRYSTALLOGRAPHY

Volume 54 (2021)

Supporting information for article:

anaklasis: a compact software package for model-based analysis of specular neutron and x-ray reflectometry data sets

Alexandros Koutsioubas

SI0 *list* structure for model definition

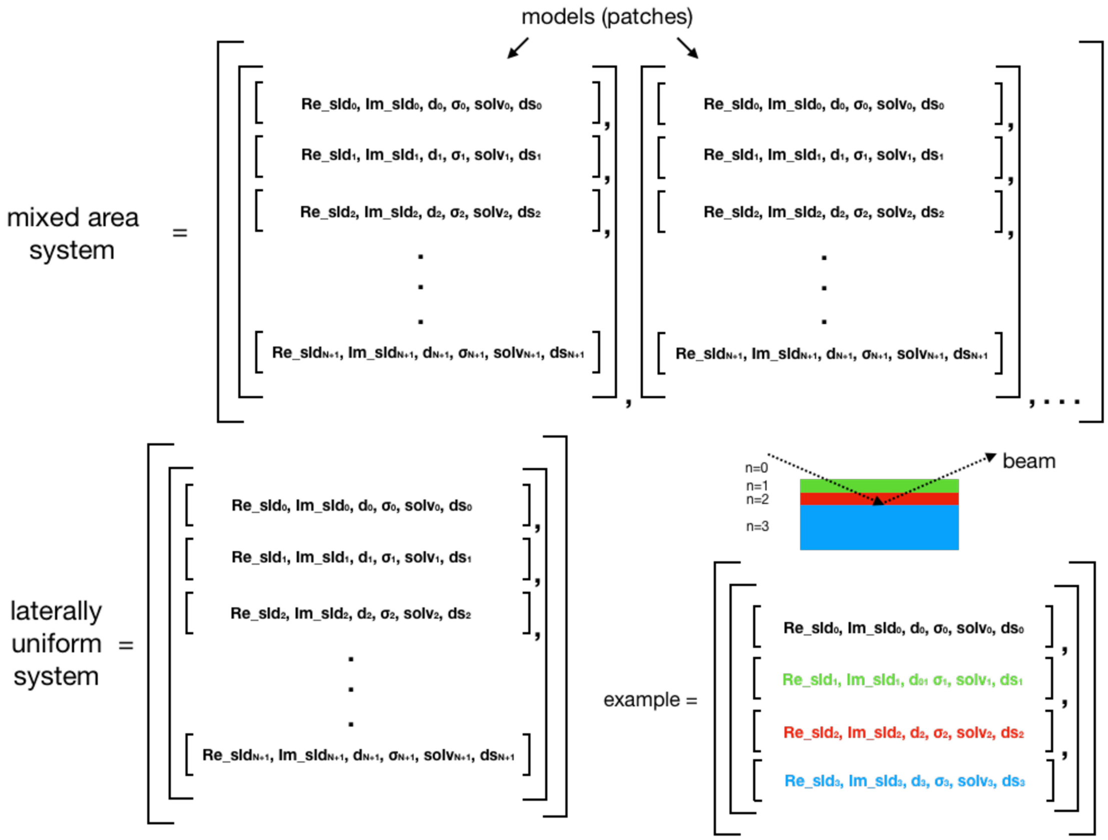

Figure SI1: Pictorial representation of *Python list* hierarchical structure (system/model/layer) for the definition of interfacial systems in *anaklasis*. The top *list* structure represents the general case of an interface containing multiple models (patches) that contribute incoherently to the reflectivity. However the most usual case is that of a laterally uniform system (single patch) as shown at the lower left. A simple example of a laterally uniform two-layer system definition is shown in the lower right. Note that the layers $n = 0$ and $n = 3$ represent the semi-infinite fronting and backing mediums respectively. Each layer contains six elements i.e. real part of sld (Re_sld), imaginary part of sld (Im_sld), thickness (d), roughness (σ), solvent volume fraction (solv) and a string description (ds). As described in the main text, the first five elements of the layer list can be either numerical values or strings containing *SymPy* mathematical expressions involving an arbitrary number of user defined parameters.

SI1 Comparison of NR data with a supported membrane model

With the code below, we calculate the NR from a supported bilayer at the Si/D_2O interface and perform a comparison with acquired reflectivity data at the MARIA neutron reflectometer (MLZ) (Mattauch *et al.*, 2018). The script structure is essentially the same as when we perform theoretical calculations with the addition of two lines specifying the input data and the units of Q.

```
from anaklasis import ref

project='membrane_ref_data_comparison'

input_file = 'membrane.dat' # input curve
units = ['A'] # Q units in Angstrom

# create a single model containing
# fronting/backing mediums and the
# 5 layers SiO2/water/heads/tails/heads
model = [
        # Re_sld   Im_sld  thk   rough  solv   description
        [  2.07e-6, 0.0, 0,     1.09, 0.0,   'Si'],
        [  3.5e-6,  0.0, 12.0, 3.50, 0.0,   'SiO2'],
        [  6.15e-6, 0.0, 3.6,  'p0', 1.0,   'D2O'],
        [  1.7e-6,  0.0, 10.5, 'p0', 0.30, 'heads'],
        [ -0.4e-6,  0.0, 30.9, 'p0', 0.03, 'tails'],
        [  1.7e-6,  0.0, 6.3,  'p0', 0.20, 'heads'],
        [  6.15e-6, 0.0,  0,   0.0,  1.0,   'D2O'],
        ]

patches=[1.0] # single patch 100% coverage
system=[model] # add single model to system list

global_param = [
        ['p0', 3.6, 'roughness'],
        ]

resolution=[-1] # pointwise resolution
background = [5.3e-7] # instrumental background
scale = [1.05] # small scale correction
qmax = [0.25]

res = ref.compare(project, input_file, units, resolution,
        patches, system, global_param,background, scale, qmax,
        experror=True, plot=True)
```

Listing SI1: *Python* code for NR data comparison with a model of a supported bilayer at the Si/D_2O interface.

Note that fronting and backing media have infinite thickness and in the script we have used the convention of inserting a zero value (although any other inserted numerical value will not influence the calculations).

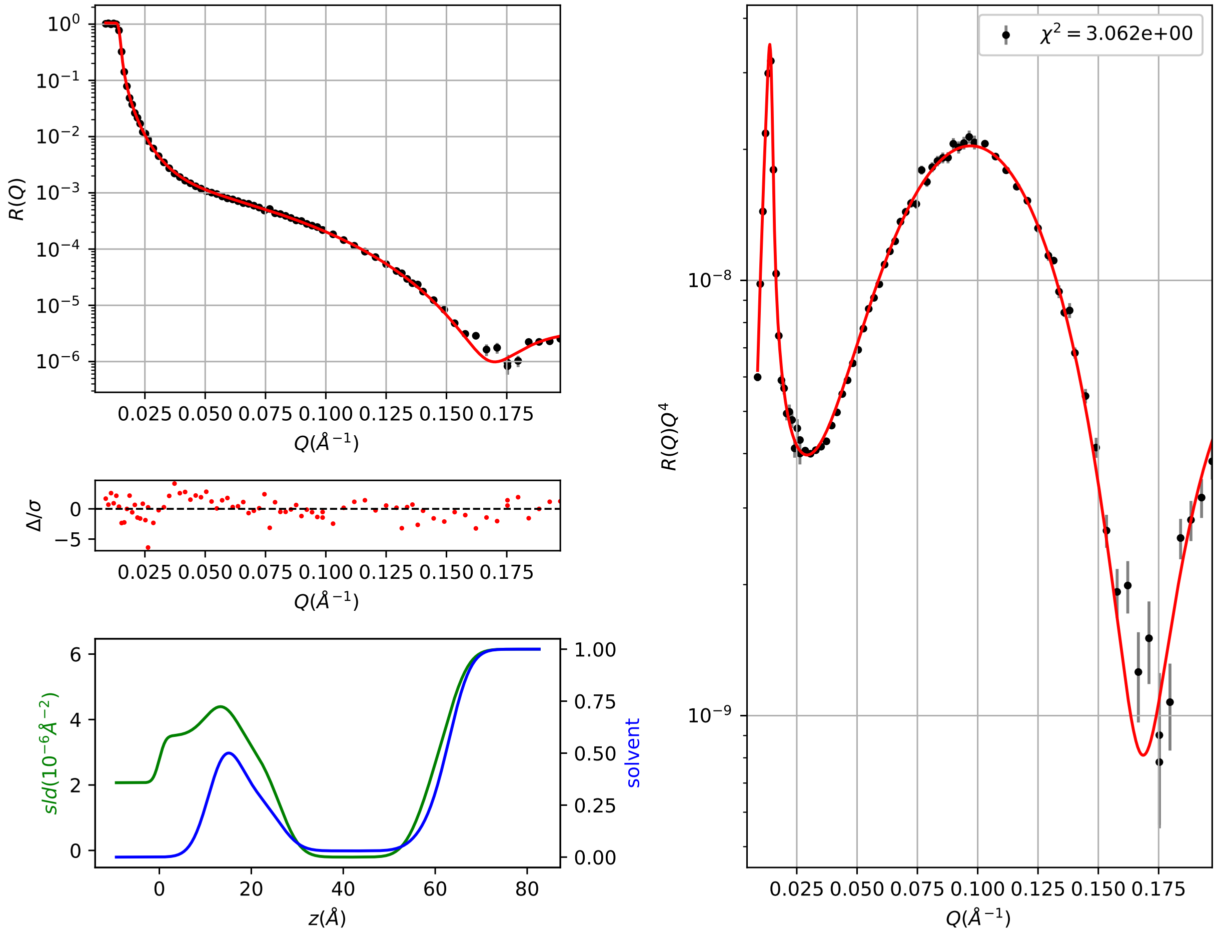

Figure SI2: NR data comparison with the theoretical reflectivity of a supported bilayer at the Si/D_2O interface.

SI2 NR calculations for a phospholipid multi-bilayer

Consider that we want to calculate the expected neutron reflectivity for a stack of ten phospholipid bilayers stacked at the Si/water(D_2O) interface. We first create the *system list* containing the layer information for the semi-infinite fronting (Si), 10 Å SiO_2, thin water layer and semi-infinite backing D_2O. Then using a *for loop* we append after the thin water layer (layer #2), ten bilayers that consist of four layers i.e. inner heads/ tails / outer heads / D_2O gaps between bilayers. So in total the *model list* contains $4 + 4 \times 10 = 44$ layers.

```
ffrom anaklasis import ref

project=’lipid_multilayer’

# Create model list where
# we first include only the fronting Si medium
# and the SiO2 and water thin layer on Si
model=[
        # Re_sld Im-sld thk rough solv description
        [  2.07e-6, 0.0, 0,  ’p0’, 0.0, ’Si’],
        [  3.50e-6, 0.0, 10, ’p0’, 0.1, ’SiO2’],
        [  6.35e-6, 0.0, 5,  ’p0’, 1.0, ’D2O␣thin␣layer’],
        ]

# "for loop" for appending bilayers on top of Si/SiO2/thin water layers
```

```
for i in range(10): # i runs from 0 to 9
                     # Re_sld Im-sld thk rough solv  description
        model.append([ 1.7e-6,  0.0, 10, 'p0', 0.3, 'inner_heads'+str(i+1)])
        model.append([ -0.5e-6, 0.0, 35, 'p0', 0.0, 'tails'+str(i+1)])
        model.append([ 1.7e-6,  0.0, 10, 'p0', 0.3, 'outer_heads'+str(i+1)])
        model.append([ 6.35e-6, 0.0, 15, 'p0', 1.0, 'D2O␣gap'+str(i+1)])

# Finally add the D2O semi-infinite backcking
model.append([  6.35e-6, 0.0, 0,  0.0,  1.0, 'D2O␣bulk'])

patches = [1.0] # single laterally uniform layer
system=[model] # we have a single model

global_param = [
        ['p0', 2.5, 'roughness'],
        ]

resolution=[0.012]
background = [5e-7]
scale = [1.0]
qmax = [0.3]

# Before calling the calculate function, one may use print(model) in order
# to check model definition
print(model)

res = ref.calculate(project,resolution,patches,system,global_param,background,scale,qmax,
    True)
```

Listing SI2: *Python* code for NR calculations concerning a lipid multi-layer at the Si/D_2O interface.

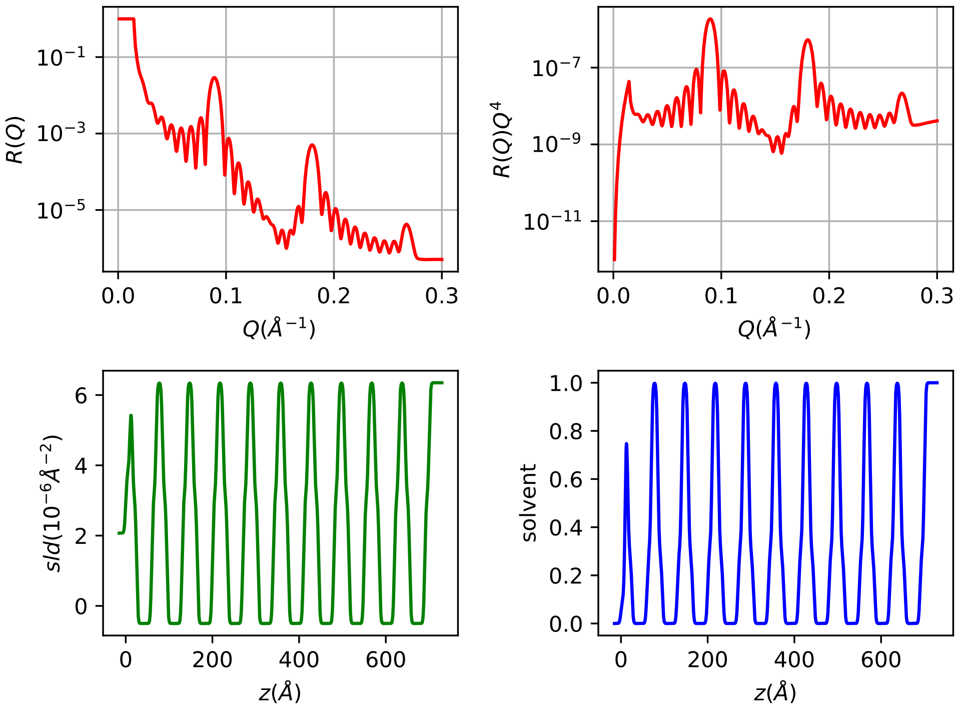

Figure SI3: NR curves for a lipid multi-layer at the Si/D_2O interface. Associated sld/solvent volume profiles are also plotted.

SI3 NR calculations for a bimodal polymer brush

Let us consider the case of a bimodal polymer brush i.e. end-grafted polymer chains at a surface. Bimodality refers to the case where two different chain length polymers are grafted with a certain grafting density. In that case the volume fraction profile of the polymer as a function of the distance from the surface (z) can be approximated by a sum of two parabolic profiles (Anastassopoulos *et al.*, 2013) as:

$$\phi(z) = max\left\{\phi_1(0)\left[1-\left(\frac{z}{L_1}\right)^2\right], 0\right\} + max\left\{\phi_2(0)\left[1-\left(\frac{z}{L_2}\right)^2\right], 0\right\} \quad \text{(SI1)}$$

where L_1, L_2 are the extensions and ϕ_1, ϕ_2 the volume fractions of the two brushes, respectively. Note that with the use of the max function we avoid negative values for z larger than the extension of the brushes. If we assume that $L_2 > L_1$ we may approximate the entire brush layer as a set of 50 slabs of thickness $L_2/50$, with an sld equal to the polymer sld and solvent volume fraction profile given by $1 - \phi(z)$. In the following code listing we construct such a brush model, where we additionally set the roughness of the slabs to $L_2/100$ in order to smooth the overall profile.

```
from anaklasis import ref

project='Bimodal_Brush'

# Create model list with just the Si/SiO2 initially
model=[
         # Re_sld Im-sld thk rough solv description
        [  2.07e-6, 0.0,   0,   3,   0.0, 'Si'],
        [  3.5e-6,  0.0,  10,   3,   0.0, 'SiO2'],
        ]

# We define the expression for the solvent volume fraction within the brush layer
expr='1-Max(p1-(p1/(p2**p5))*((p4/50.0)*(n-1.5))**p5,0)-p3+(p3/(p4**p6))*((p4/50.0)*(n-1.5))
    **p6'

# we append 50 "brush" slabs after the SiO2 layer.
                                # Re_sld Im-sld   thk        rough       solv  description
for i in range(50): model.append([ 'p7', 0.0, 'p4/50.0', 'p4/100.0', expr, 'brush_layer'])

# Finally appaend the semi-infinite backing (D2O)
model.append([  'p0',    0.0,  0,   0,   1.0, 'D2O'])

patches = [1.0] # single laterally uniform layer
system=[model] # Note we habe a single model(patch)

global_param = [
    # name value  description
        ['p0', 6.35e-6, 'solvent␣sld'],
        ['p1', 0.4, 'brush1_phi0'],
        ['p2', 400, 'brush1_length'],
        ['p3', 0.2, 'brush2_phi0'],
        ['p4', 900, 'brush2_length'],
        ['p5', 2, 'exponent_brush1'],
        ['p6', 2, 'exponent_brush2'],
        ['p7', 0.7e-6, 'polymer_sld'],
        ]

resolution=[0.07] # dQ/Q=7%
background = [0.0] # no background
scale = [1.0]
qmax = [0.1]

res = ref.calculate(project,resolution,patches,system,global_param,background,scale,qmax,
    True)
```

Listing SI3: *Python* code for NR calculations from a polystyrene bimodal polymer brush at the Si/d-toluene interface.

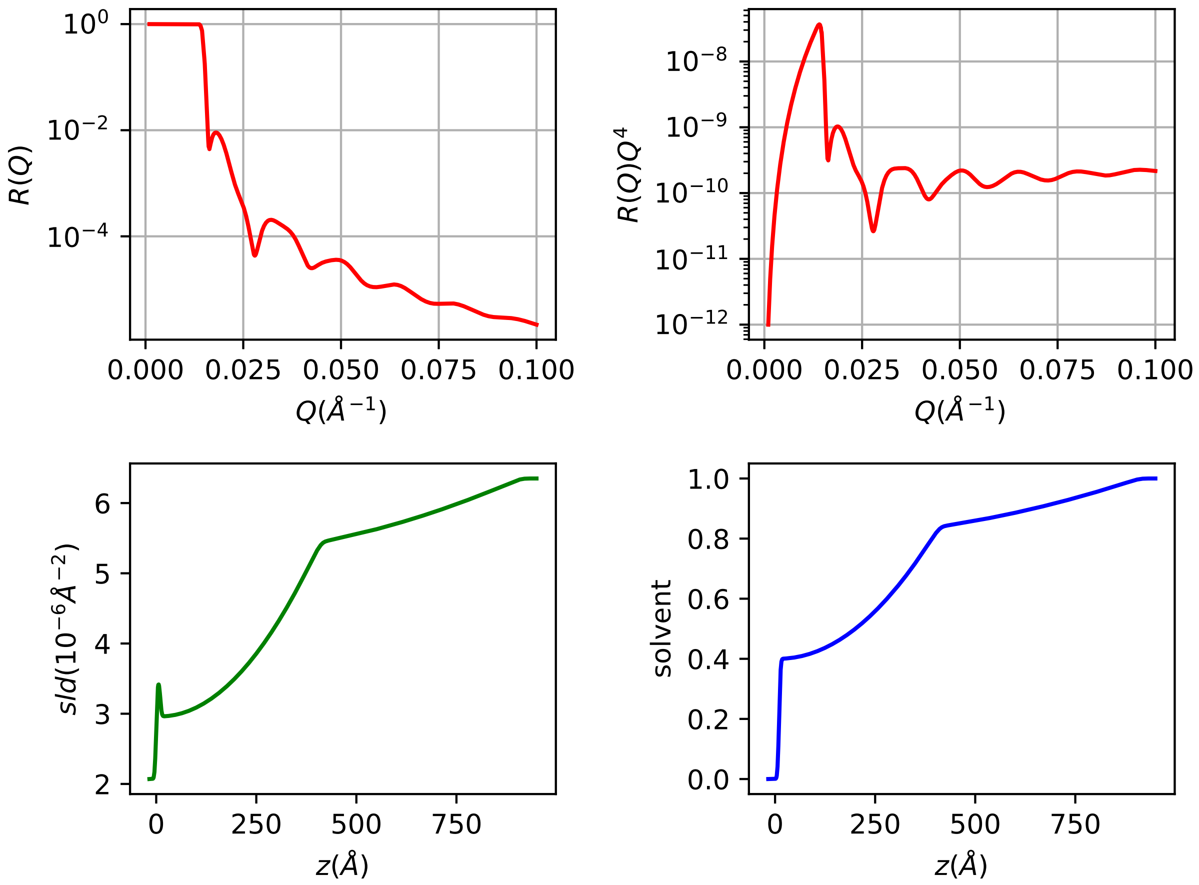

Figure SI4: NR curves and sld/solvent volume profiles for a bimodal polystyrene brush at the Si/d-toluene interface.

SI4 Polydisperse nanoparticle islands

Here we calculate the reflectivity for a system of polydisperse nanoparticle islands at the solid/liquid interface. As discussed in the main manuscript, assuming that the nanoparticle diameter is distributed normally, the solvent volume fraction of the nanoparticle layer is given by:

$$1 - \phi(z) \approx \int_{D-3\sigma_D}^{D+3\sigma_D} f(x) Min \left[1 - \frac{4A}{x^2}\left(xz - z^2\right), 1\right] dx \tag{SI2}$$

where

$$f(x) = \frac{1}{\sigma_D\sqrt{2\pi}} \exp\left[-\frac{1}{2}\left(\frac{x-D}{\sigma_D}\right)^2\right] \tag{SI3}$$

we may approximate the integral by a sum of the form:

$$1 - \phi(z) \approx \sum_{i=-30}^{i=30} f(D + i\sigma_D/10) \cdot min \left[1 - \frac{4A}{(D + i\sigma_D/10)^2}\left((D + i\sigma_D/10)z - z^2\right), 1\right] (\sigma_D/10) \tag{SI4}$$

with *SymPy* we may construct an expression based on the above sum using one of the three available summation variables used in *anaklasis* (ii, jj, kk). Note that the use of the *min* function ensures that no negative values contribute to the sum.

```python
# reflectivity calculations for polydisperse nanoparticle islands
from anaklasis import ref

project='nanoparticle_islands'

# model0 Si/D2O interface
model0=[
        #  Re_sld   Im_sld thk rough solv decription
        [ 2.07e-6,  0.0e-6, 0, 2.0,  0.0, 'Si'],
        [ 6.35e-6,  0.0e-6, 0, 0.0,  1.0, 'D2O'],
        ]

# model1 Si/nanoparticles/D2O
# First we declare just Si semi-infinite fronting
model1=[
        #  Re_sld   Im_sld thk rough solv decription
        [ 2.07e-6,  0.0e-6, 0,  2.0, 0.0, 'Si'],
        ]

# Approximate solvent volume fraction integral using a sum.
expr="""Sum(((1/(p2*(2*pi)**0.5))*exp(-0.5*(((p1+(ii/10)*p2)-p1)/p2)**2))*
Min(1-(4*p0/(p1+(ii/10)*p2)**2)*((p1+(ii/10)*p2)*(n-0.5)*(p1/100)-((n-0.5)*(p1/100))**2),1)
*(p2/10),(ii,-30,30))"""

# Define solvent volume fraction for each NP slice. Note that n is the layer number.
# The product of (n-0.5) x slice thickness gives us the middle z point of each slice.

# append NP layer sliced in 170 slabs.
for i in range(170):
        model1.append([ 1.41e-6, 0.0e-6, 'p1/100', 0.0, expr, 'NP_layer'])

# Finally append D2O semi-infinite backing in model1
model1.append([ 6.35e-6,  0.0e-6, 0,  0.0, 1.0, 'D2O'])

# Define different patch coverages that should add up to unity
patches=[0.3,0.7]
system=[model0,model1] # Note we have two models

global_param = [
        ['p0', 0.91, 'packing_constant'],
        ['p1', 150, 'nanoparticle_diameter'],
        ['p2', 40, 'nanoparticle_diameter_sd'],
        ]

resolution=[0.05]
background = [1.0e-7]
scale = [1.0]
qmax = [0.3]

res = ref.calculate(project, resolution, patches,
        system, global_param,
        background,scale, qmax, plot=True)
```

Listing SI4: *Python* code for NR calculations from a film of polydisperse nanoparticle islands at the Si/D_2) interface.

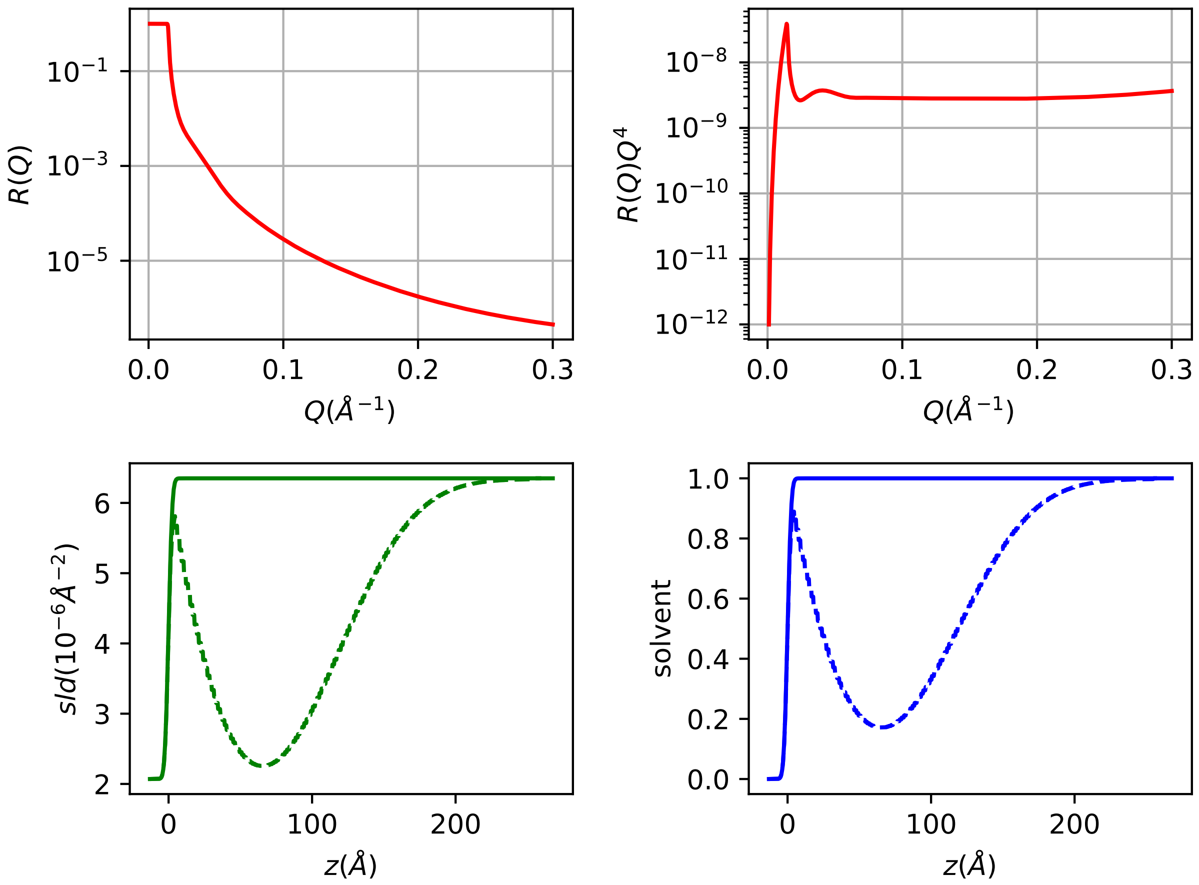

Figure SI5: NR curves and sld/solvent volume profiles for polydisperse nanoparticle islands at the solid/liquid interface. In the profile plots, full lines correspond to the Si/D_2O model and dotted lines to the Si/nanoparticles/D_2O model.

-

SI5 Refinement code and corner plot of three-solvent-contrast supported-lipid-bilayer refinement

```
from anaklasis import ref

project='supported_bilayer_refinement'
in_file=['bilayer_D2O.dat','bilayer_SMW.dat','bilayer_H2O.dat']
units=['A','A','A'] # all units in Angstrom
fit_mode=0 # 0 is for linear, 1 is for log
fit_weight=[1,1,1] # equal fit weight for all three curves
method = 'mcmc' # perform MCMC
model = [
        # Re_sld Im_sld thk rough solv description
        [  2.07e-6, 0.0, 0, 2.0, 0.0, 'Si'],
        [ 3.5e-6, 0.0, 10, 'p0', 0.0, 'SiO2'],
        [ 'm0', 0.0, 'p1', 'p0', 1.0, 'water'],
        [ 'p5/(p9*p3)', 0.0, 'p3', 'p0', '1.0-p7/(p9*p3)', 'inner_heads'],
        [ 'p6/(p9*p2)', 0.0, 'p2', 'p0', '1.0-p8/(p9*p2)', 'inner_tails'],
        [ 'p6/(p9*p2)', 0.0, 'p2', 'p0', '1.0-p8/(p9*p2)', 'outer_tails'],
        [ 'p5/(p9*p4)', 0.0, 'p4', 'p0', '1.0-p7/(p9*p4)', 'outer_heads'],
        [  'm0', 0.0,  0, 0.0, 1.0, 'bulk'],
        ]
patches=[1.0]
system=[model]
param = [
    # param  min  max  description, for type 'uniform'
    # param  mean sd   description  for type 'normal'
        ['p0', 2.0, 0.5, 'roughness','normal'],
        ['p1', 3.0, 15.0, 'water_d','uniform'],
        ['p2', 7.0, 20.0, 'tail_d','uniform'],
        ['p3', 5.0, 15.0, 'inner_head_d','uniform'],
        ['p4', 5.0, 15.0, 'outer_head_d','uniform'],
        ['p5', 6.01e-4, 6.01e-4, 'b_heads','uniform'],
        ['p6', -2.92e-4, -2.92e-4, 'b_tails','uniform'],
        ['p7', 319, 319, 'V_heads','uniform'],
        ['p8', 782, 782, 'V_tails','uniform'],
        ['p9', 40, 70, 'area_lipid','uniform'],
        ]
# Note the multiparameter m0 represents the solvent sld that assumes a different
# set of bounds for each input curve.
multi_param = [
        # param  min  max  min max min max description type
        ['m0', 6.15e-6, 6.40e-6, 1.80e-6, 2.30e-6, -0.56e-6, 0.0e-6, 'solv_sld','uniform']
        ]
constraints = [
        '1.0-p8/(p9*p2)>0', # With these three constaints we avoid that the solvent volume
        '1.0-p7/(p9*p4)>0', # fraction might become negative for the inner_head, inner_tail
        '1.0-p7/(p9*p3)>0', # outer_tail ot outer_head layer.
        ]
resolution=[-1,-1,-1] # pointwise resolution
background = [
        [0.0,1.0e-5,'uniform'], # background is left free for all three curves
        [0.0,1.0e-5,'uniform'],
        [0.0,1.0e-5,'uniform'],
        ]
scale = [
        [1.0,0.2,'normal'], # curve scale is also a free parmater for all curves
        [1.0,0.2,'normal'],
        [1.0,0.2,'normal'],
        ]
res = ref.fit(project,in_file,units,fit_mode,fit_weight,method,resolution,patches,system,
        param,multi_param,constraints,background,scale,experror=True,plot=True,fast=True)
```

Listing SI5: Commented *Python* code for three-contrast NR refinement from a supported lipid bilayer at the Si / water interface.

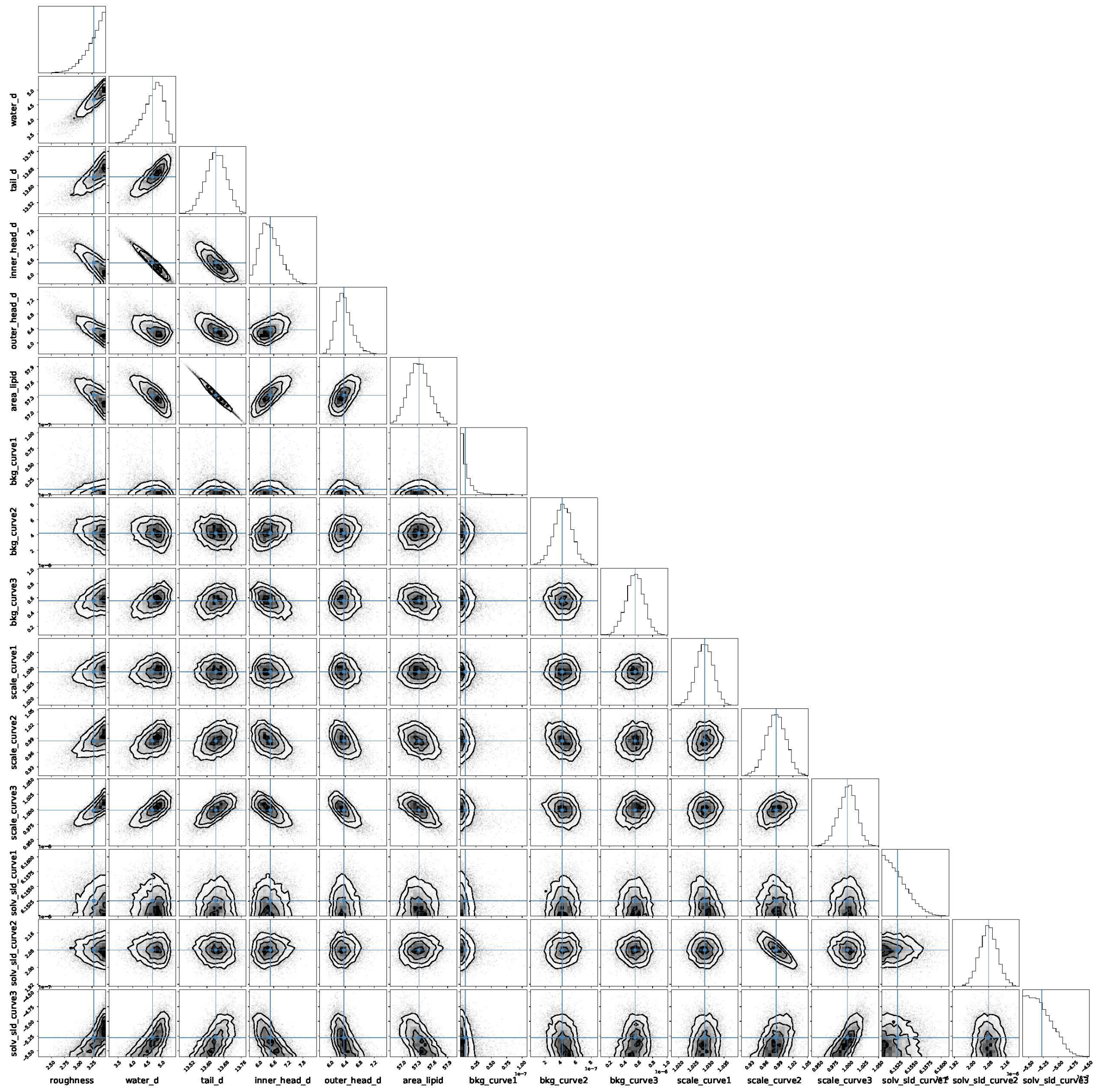

Figure SI6: Corner plot of the free parameters from a supported-bilayer three-solvent-contrast-data refinement.The panels on the diagonal show the 1-D histogram for each model parameter obtained by marginalising over the other parameters, with a blue line to indicate the mean value. The off-diagonal panels show 2-D projections of the posterior probability distributions for each pair of parameters.

SI6 Co-refinement of seven NR curves from a lipid monolayer at the air/water interface

In this section we co-refine neutron reflectivity data of a DSPC lipid monolayer at the air / water interface and at a surface pressure of 30 mN m^{-1}, acquired by (Hollinshead *et al.*, 2009) and thoroughly reanalysed by (McCluskey *et al.*, 2019, 2020). The input data are:

- "hd2o30.dat" - DSPC in D_2O
- "d13d2o30.dat" - head-deuterated DSPC in D_2O
- "d13acmw30.dat" - head-deuterated DSPC in air-matched water (sld=0.0)
- "d70d2o30.dat" - tail-deuterated DSPC in D_2O
- "d70acmw30.dat" - tail-deuterated DSPC in air-matched water (sld=0.0)
- "d83d2o30.dat" - fully-deuterated DSPC in D_2O
- "d83acmw30.dat" - fully-deuterated DSPC in air-matched water (sld=0.0)

We model the interface between air and water using a two-layer model as lipid tails / lipid heads, where solvent may partially penetrate only the head layer (so that the tail volume fraction $\phi_t = 1$). Since the number density of the head and tail components is the same, we have for the head-layer volume fraction (McCluskey *et al.*, 2019):

$$\phi_h = \frac{d_t V_h}{V_t d_h} \tag{SI5}$$

where t is the layer thickness and V the molecular volume, with subscript h and t corresponding to heads and tails respectively. The sld of the head and tail layer is given by $\phi_h b_h / V_h$ and $\phi_t b_t / V_t$, where b is the scattering length. In the following listing, we use multi-parameters in order to set the scattering lengths of heads and tails and the sld of the solvent for each one of the seven input curves. We also use the above expressions for setting the sld and volume fraction profile of the two layers. An inequality is added in the constraints *list* ensuring that $\phi_h > 0$ during the refinement. The free parameters of the model are the overall roughness, V_t, V_h, d_t, d_h and the background and scale of the seven curves, adding up to a total of 19 parameters.

```
from anaklasis import ref

project='lipid_monolayer'

# import seven experimental curves
in_file=['hd2o30.dat','d13d2o30.dat','d13acmw30.dat','d70d2o30.dat','d70acmw30.dat',
                'd83d2o30.dat','d83acmw30.dat']

# all units in Angstrom
units=['A','A','A','A','A','A','A']

fit_mode=0 # use linear Figure of Merit

# all curves equal fit weight
fit_weight=[1,1,1,1,1,1,1]

method = 'mcmc' # perform mcmc

# dQ/Q = 10% for all input curves

resolution=[0.1,0.1,0.1,0.1,0.1,0.1,0.1]
model= [
        # Re_sld Im_sld thk rough solv description
        [  0.0e-6, 0.0, 0, 'p0', 0.0, 'Air'],
        [ 'm2*p4/p5', 0.0, 'p3', 'p0', '1.0-p4', 'tails'],
        [ 'm1*((p3*p2*p4)/(p5*p1))/p2', 0.0, 'p1', 'p0', '1.0-(p3*p2*p4)/(p5*p1)', 'heads'],
```

```
        [  'm0', 0.0,  0, 0.0, 1.0, 'bulk'],
        ]

patches=[1.0] # single uniform layer
system=[model]

# Definition of Global parameters
param = [
    # param  min  max  description
        ['p0', 3.0, 5.0, 'roughness','uniform'],
        ['p1', 8.0, 16.0, 'heads␣thickness','uniform'],
        ['p2', 300, 380.0, 'head␣volume','uniform'],
        ['p3', 10.0, 26.0, 'tail␣thickness','uniform'],
        ['p4', 1.0, 1.0, 'tail␣vol␣fraction','uniform'],
        ['p5',800, 1000, 'tail␣volume','uniform'],
        ]

# Definition of multi parameters
# solvent sld, lipid head and tail scattering lengths
multi_param = [
        # param  min  max  min max ... description
        ['m0', 6.35e-6, 6.35e-6, 6.35e-6, 6.35e-6, -0.00e-6, 0.00e-6,6.35e-6, 6.35e-6,
         -0.00e-6, 0.00e-6,6.35e-6, 6.35e-6, -0.00e-6, 0.00e-6, 'solvent_sld','uniform'],
        ['m1', 6.01e-4, 6.01e-4, 19.54e-4, 19.54e-4, 19.54e-4, 19.54e-4, 11.21e-4, 11.21e-4,
         11.21e-4, 11.21e-4, 24.75e-4, 24.75e-4, 24.75e-4, 24.75e-4 ,'b_heads','uniform'],
        ['m2', -3.58e-4, -3.58e-4, -3.58e-4, -3.58e-4, -3.58e-4, -3.58e-4, 69.32e-4, 69.32e
            -4,
         69.32e-4, 69.32e-4, 69.32e-4, 69.32e-4, 69.32e-4, 69.32e-4 ,'b_tails','uniform'],
        ]

# Solvent volume fraction should stay always
# larger than zero
constraints = [
        '(p3*p2*p4)/(p5*p1)<1',
        ]
# background as free parameter
background = [
        [0.0e-7,4.0e-6,'uniform'],
        [0.0e-7,4.0e-6,'uniform'],
        [0.0e-7,4.0e-6,'uniform'],
        [0.0e-7,4.0e-6,'uniform'],
        [0.0e-7,4.0e-6,'uniform'],
        [0.0e-7,4.0e-6,'uniform'],
        [0.0e-7,4.0e-6,'uniform'],
        ]
# curve scale as a free parameter
scale = [
        [0.10,0.25,'uniform'],
        [0.10,0.25,'uniform'],
        [0.10,0.25,'uniform'],
        [0.10,0.25,'uniform'],
        [0.10,0.25,'uniform'],
        [0.10,0.25,'uniform'],
        [0.10,0.25,'uniform'],
        ]

res = ref.fit(project, in_file, units, fit_mode, fit_weight, method, resolution,
                         patches, system, param, multi_param, constraints,background,scale,
                         experror=True,plot=True,fast=True)
```

Listing SI6: Commented *Python* code for seven-contrast NR refinement from a DSCP lipid monolayer at the air / water interface.

The refinement gave the following parameter values, $\sigma = 3.15 \pm 0.12$ Å, $d_h = 8.70 \pm 0.08$ Å, $d_t = 20.0 \pm 0.2$ Å, $V_h = 352 \pm 6$ Å^3 and $V_t = 913 \pm 8$ Å^3. Through MCMC sampling we also obtain a corner plot where we see no appreciable covariance between any pairs of the parameters.

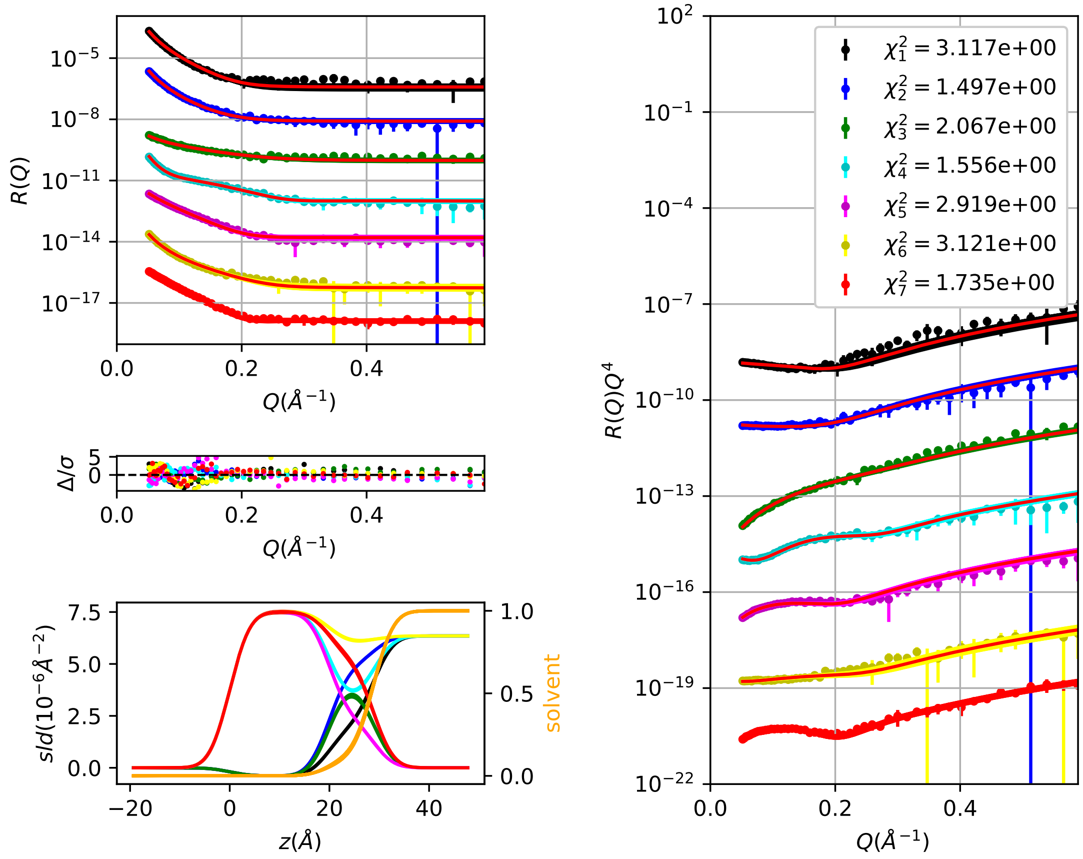

Figure SI7: Fitted NR data (seven different contrasts) and sld/solvent volume fraction profiles for a lipid monolayer at the air/water interface. black,blue,green,cyan,magenta,yellow,red colours correspond to DSPC in D_2O, head-deuterated DSPC in D_2O, head-deuterated DSPC in air-matched water, tail-deuterated DSPC in D_2O, tail-deuterated DSPC in air-matched water, fully deuterated DSPC in D_2O, fully-deuterated DSPC in air-matched water, respectively.

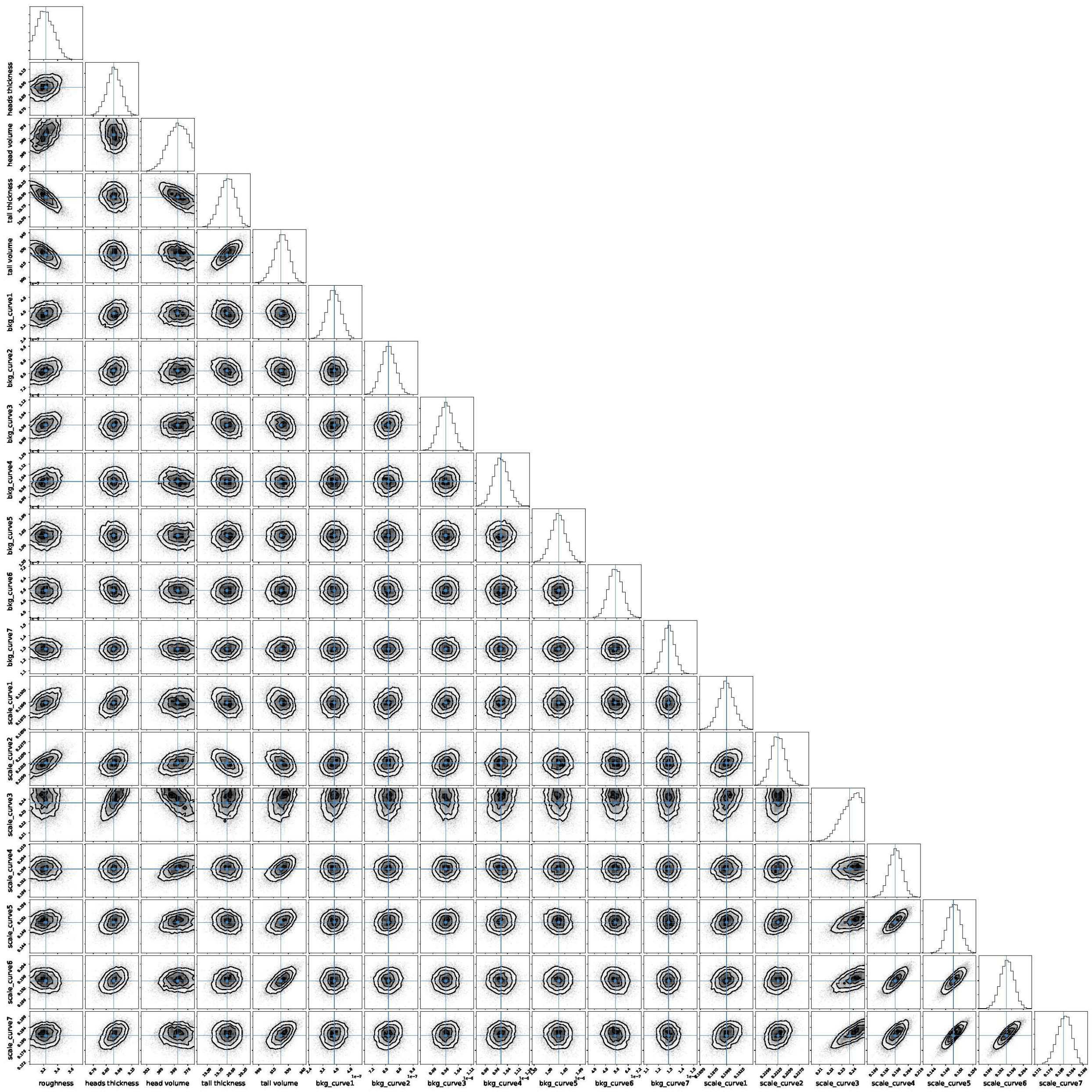

Figure SI8: Corner plot of the free parameters from a lipid monolayer at the air/water interface, seven-contrast NR data refinement.

SI7 Refinement of PNR data

```python
from anaklasis import ref

project='PNR_FeNi'
in_file=[]
in_file.append('FeNi_Au_ud.dat')
in_file.append('FeNi_Au_dd.dat')

units=['A','A'] # all input Q in inverse Angstrom

fit_mode=0 # 0 is linear, 1 is log
fit_weight=[1.0,1.0] # equal fit weight for both curves
method = 'simple' # can be 'simple', or 'mcmc' or 'bootstrap'

# Here we define the model Si/SiO2/FeNi/Au/D2O
# note the expression for the sld of the FeNi layer
# nuclear sld plus or minus magnetic sld
model = [
        # Re_sld Im_sld thk rough solv description
        [  2.07e-6, 0.0, 0, 2.0, 0.0, 'Si'],
        [ 3.5e-6, 0.0, 12, 2.0, 0.0, 'SiO2'],
        [ 'p0+m0*p1', 0.0, 'p2', 'p4', 0.0, 'FeNi'],
        [ 4.66e-6, 0.0, 'p3', 'p5', 0.0, 'Au'],
        [  'p6', 0.0,  0, 0.0, 1.0, 'D2O'],
        ]
patches=[1.0]
system=[model]
model_param = [
    # param  min  max  description type
        ['p0', 7.57e-6, 9.57e-6, 'nuclear_sld','uniform'],
        ['p1', 1.5e-6, 5e-6, 'magnetic_sld','uniform'],
        ['p2',100,700,'FeNi_thickness','uniform'],
        ['p3',50,150,'Au_thickness','uniform'],
        ['p4',0,20,'FeNi_roughness','uniform'],
        ['p5',0,20,'Au_roughness','uniform'],
        ['p6',6.0e-6,6.4e-6,'solvent_sld','uniform'],
        ]
# we define a multiparameter (m0) that assumes a value -1 or +1
# for subtracting or adding the magnetic sld to the total
# sld depending on the polarsation of the incoming beam
multi_param = [
        # param  min  max  min max ... description
        ['m0',  -1.0, -1.0, 1.0, 1.0, 'up/down','uniform']
        ]

constraints = [] # no constraints
resolution=[0.1,0.1] # dQ/Q=10%

# background corrected data so evrything set to zero
background = [
        [0.0,0.0,'uniform'],
        [0.0,0.0,'uniform'],
        ]
# scaling of the curve left slightly free
scale = [
        [1.0,1.0,'uniform'],
        [1.0,1.0,'uniform'],
        ]

res = ref.fit(project, in_file, units, fit_mode, fit_weight, method, resolution, patches,
    system,
        model_param, multi_param, constraints,background,scale,experror=False,plot=True,fast
            =False)
```

Listing SI7: Commented *Python* code for a fit of polarized NR data from Fe-Ni alloy/Au at the silicon/D_2O interface.

SI8 Co-refinement of XRR and PNR data

A final example concerns the co-refinement of XRR and PNR data from the same sample. We use data acquired on the MARIA reflectometer (MLZ) (Mattauch *et al.*, 2018) and on an in-house x-ray reflectometer that concern a Fe/Pt layer pair on an MgO substrate. As can be seen in the following code listing, declaration of the model is quite simple for this two-layer system. We use a multi-parameter ($m4$) for the magnetic sld contribution (applied magnetic field during NR measurement 0.1 Tesla). For XRR the contribution is zero, and depending on the spin orientation of the beam, ± 1 for the two NR curves. Since we co-refine XRR and NR data, the real and imaginary parts of the sld of each layer are different for each input curve. Consequently we use multi-parameters for setting the sld for each curve. Note that no experimental error $\delta R(Q)$ is present in the XRR data, so we cannot perform MCMC or bootstrap analysis.

```
from anaklasis import ref

project='XRR_fit'
in_file=[]
in_file.append('Fe_Pt-XRR.dat') # XRR curve
in_file.append('Fe_Pt_UP.dat')  # NR curve spin up
in_file.append('Fe_Pt_DN.dat')  # NR curve spin down

units=['A','A','A'] # all data in Angstrom units

fit_mode=0 # linear FOM
fit_weight=[1,1,1] # equal fit weight for all curves

method = 'simple' # no MCMC or Bootstrap

model = [
        # Re_sld Im_sld thk rough solv description
        [  0.0e-6, 0.0, 0, 'p1', 0.0, 'air'],
        [ 'm0', 'm1', 'p2', 'p3', 0.0, 'Pt'],
        [ 'm2+m4*p0', 'm3', 'p4', 'p5', 0.0, 'Fe'],
        [ 'm5', 'm6',  0, 0.0, 0.0, 'MgO'],
        ]

patches=[1.0]
system=[model]

param = [
    # param  min  max  description type
    ['p0', 4.5e-6, 4.5e-6, 'Fe-␣mag␣sld','uniform'],
    ['p1', 0, 6, 'air/Pt␣roughness','uniform'],
    ['p2', 20, 30, 'Pt␣thickness','uniform'],
    ['p3', 0, 6, 'Pt/Fe␣roughness','uniform'],
    ['p4', 200, 250, 'Fe␣thickness','uniform'],
    ['p5', 0, 6, 'Fe/MgO␣roughness','uniform'],
        ]

multi_param = [
        # param  min_XRR  max_XRR  min_NR_up max_NR_up min_NR_down max_NR_down  description
            type
        ['m0', 13.7e-5, 13.7e-5, 6.35e-6, 6.35e-6, 6.35e-6, 6.35e-6, 'Pt-Re(sld)','uniform'
            ],
        ['m1', 1.35e-5, 1.35e-5, 0.0, 0.0, 0.0, 0.0, 'Pt-Im(sld)','uniform'],
        ['m2', 5.94e-5, 5.94e-5, 8.024e-6, 8.024e-6, 8.024e-6, 8.024e-6, 'Fe-nuclear␣Re(sld)
            ','uniform'],
        ['m3', 7.69e-6, 7.69e-6, 0.0, 0.0, 0.0, 0.0, 'Fe-nuclear␣Im(sld)','uniform'],
        ['m4', 0.0, 0.0, 1.0, 1.0, -1.0, -1.0, 'mag_sld␣contribution','uniform'],
        ['m5', 3.06e-5, 3.06e-5, 6.01e-6, 6.01e-6, 6.01e-6, 6.01e-6, 'MgO-Re(sld)','uniform'
            ],
        ['m6', 3.26e-7, 3.26e-7, 0.0, 0.0, 0.0, 0.0, 'MgO-Im(sld)','uniform'],
        ]

constraints = [
        ]
```

```python
resolution=[0.0, 0.12, 0.12] # dQ/Q for XRR and the two NR curves

background = [
        [0.0e-11,1.0e-5,'uniform'],
        [0.0e-11,1.0e-5,'uniform'],
        [0.0e-11,1.0e-5,'uniform'],
        ]

scale = [
        [1.5,2.5,'uniform'],
        [1.00,1.00,'uniform'],
        [1.00,1.00,'uniform'],
        ]

res = ref.fit(project, in_file, units, fit_mode,fit_weight, method, resolution, patches,
    system, param, multi_param, constraints,background,scale,experror=False, plot=True,fast=
    False)
```

Listing SI8: *Python* code for XRR/NR data co-refinement for a MgO/Fe/Pt system.

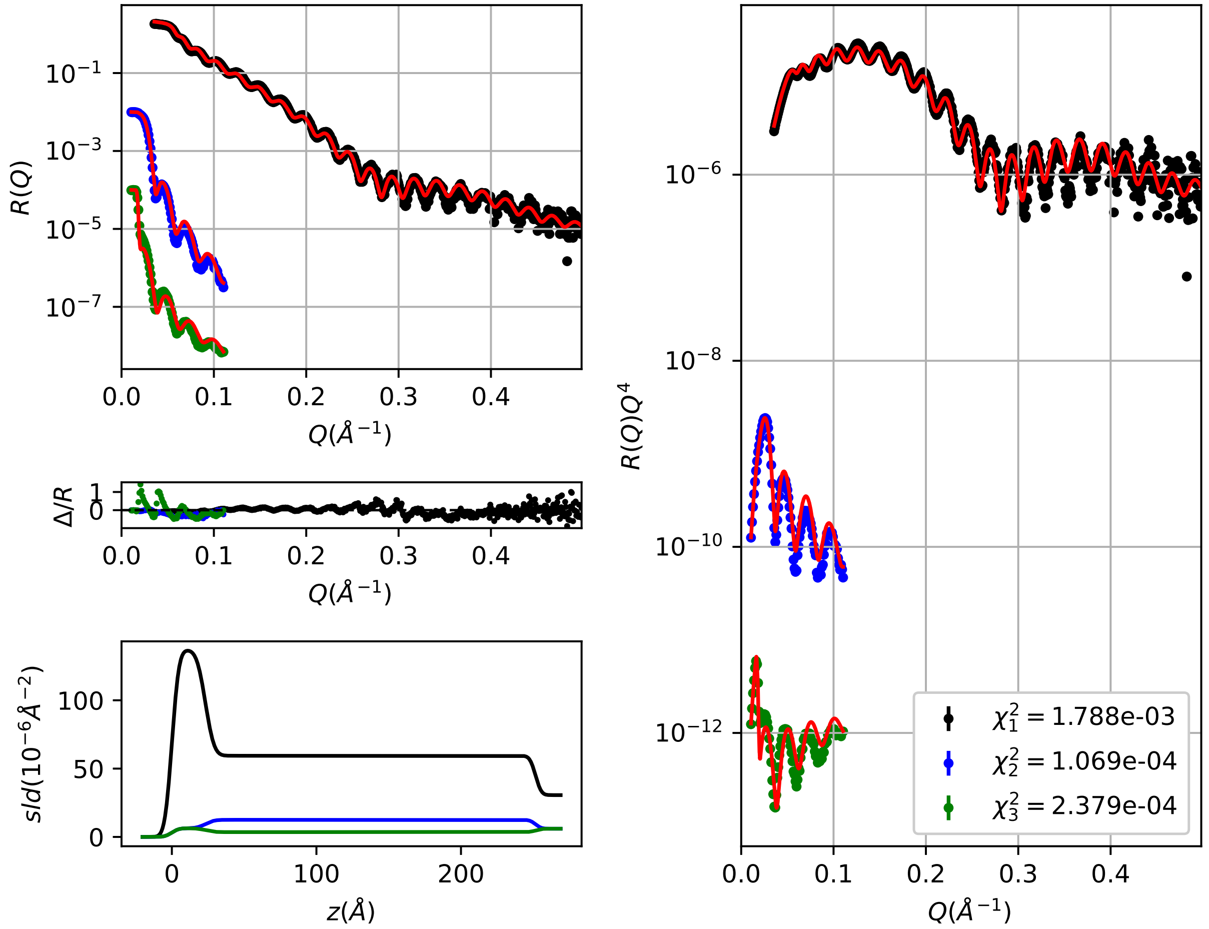

Figure SI9: Co-refined XRR/PNR data and sld profiles for a Fe/Pt pair of layers at the air/MgO interface. Black points correspond to the XRR data, blue points correspond to NR spin-up data and green points correspond to NR spin-down data.

References

Anastassopoulos, D. L., Spiliopoulos, N., Vradis, A. A., Toprakcioglu, C., Menelle, A. and Cousin, F. (2013). *Macromolecules*, 46(17), 6972–6980.

Hollinshead, C. M., Harvey, R. D., Barlow, D. J., Webster, J. R. P., Hughes, A. V., Weston, A. and Lawrence, M. J. (2009). *Langmuir*, 25(7), 4070–4077.

Mattauch, S., Koutsioubas, A., Rücker, U., Korolkov, D., Fracassi, V., Daemen, J., Schmitz, R., Bussmann, K., Suxdorf, F., Wagener, M., Kämmerling, P., Kleines, H., Fleischhauer-Fuß, L., Bednareck, M., Ossoviy, V., Nebel, A., Stronciwilk, P., Staringer, S., Gödel, M., Richter, A., Kusche, H., Kohnke, T., Ioffe, A., Babcock, E., Salhi, Z. and Bruckel, T. (2018). *Journal of Applied Crystallography*, 51(3), 646–654.

McCluskey, A. R., Cooper, J. F. K., Arnold, T. and Snow, T. (2020). *Machine Learning: Science and Technology*, 1(3), 035002.

McCluskey, A. R., Grant, J., Smith, A. J., Rawle, J. L., Barlow, D. J., Lawrence, M. J., Parker, S. C. and Edler, K. J. (2019). *Journal of Physics Communications*, 3(7), 075001.
